# Management of congenital nephrotic syndrome: consensus recommendations of the ERKNet-ESPN Working Group

**DOI:** 10.1038/s41581-020-00384-1

**Published:** 2021-01-29

**Authors:** Olivia Boyer, Franz Schaefer, Dieter Haffner, Detlef Bockenhauer, Tuula Hölttä, Sandra Bérody, Hazel Webb, Marie Heselden, Beata S. Lipska-Zie˛tkiewicz, Fatih Ozaltin, Elena Levtchenko, Marina Vivarelli

**Affiliations:** 1 Department of Pediatric Nephrology, Reference center for Idiopathic Nephrotic Syndrome in Children and Adults, Imagine Institute, Paris University, Necker Hospital, APHP, Paris, France; 2 Laboratory of Hereditary Kidney Diseases, Imagine Institute, INSERM U1163, Paris Descartes University, Paris, France; 3 Division of Pediatric Nephrology, Center for Pediatrics and Adolescent Medicine, Heidelberg, Germany; 4 Department of Pediatric Kidney, Liver and Metabolic Diseases, Children’s Hospital, Hannover Medical School, Hannover, Germany; 5 Center for Congenital Kidney Diseases, Center for Rare Diseases, Hannover Medical School, Hannover, Germany; 6 UCL Department of Renal Medicine and Renal Unit, Great Ormond Street Hospital for Children NHS Foundation Trust, London, UK; 7 Department of Pediatric Nephrology and Transplantation, The New Children’s Hospital, HUS Helsinki University Hospital, Helsinki, Finland; 8 Patient representative, London, UK; 9 Clinical Genetics Unit, Department of Biology and Medical Genetics, Medical University of Gdańsk, Gdańsk, Poland; 10 Centre for Rare Diseases, Medical University of Gdańsk, Gdańsk, Poland; 11 Department of Pediatric Nephrology and Nephrogenetics Laboratory, Hacettepe University Faculty of Medicine, Ankara, Turkey; 12 Division of Pediatric Nephrology, Department of Pediatrics, University Hospitals Leuven; Department of Development & Regeneration, University of Leuven, Leuven, Belgium; 13 Division of Nephrology and Dialysis, Department of Pediatric Subspecialties, Bambino Gesù Pediatric Hospital Istituto di Ricerca e Cura a Carattere Scientifico (IRCCS), Rome, Italy

**Keywords:** Paediatric kidney disease, Focal segmental glomerulosclerosis

## Abstract

Congenital nephrotic syndrome (CNS) is a heterogeneous group of disorders characterized by nephrotic-range proteinuria, hypoalbuminaemia and oedema, which manifest in utero or during the first 3 months of life. The main cause of CNS is genetic defects in podocytes; however, it can also be caused, in rare cases, by congenital infections or maternal allo-immune disease. Management of CNS is very challenging because patients are prone to severe complications, such as haemodynamic compromise, infections, thromboses, impaired growth and kidney failure. In this consensus statement, experts from the European Reference Network for Kidney Diseases (ERKNet) and the European Society for Paediatric Nephrology (ESPN) summarize the current evidence and present recommendations for the management of CNS, including the use of renin–angiotensin system inhibitors, diuretics, anticoagulation and infection prophylaxis. Therapeutic management should be adapted to the clinical severity of the condition with the aim of maintaining intravascular euvolaemia and adequate nutrition, while preventing complications and preserving central and peripheral vessels. We do not recommend performing routine early nephrectomies but suggest that they are considered in patients with severe complications despite optimal conservative treatment, and before transplantation in patients with persisting nephrotic syndrome and/or a *WT1*-dominant pathogenic variant.

## Abstract (web summary)

Here, experts from the European Reference Network for Kidney Diseases and the European Society for Paediatric Nephrology present recommendations for the management of congenital nephrotic syndrome, including the use of renin–angiotensin system inhibitors, diuretics, anticoagulation and infection prophylaxis.

## Introduction

Congenital nephrotic syndrome (CNS) is characterized by nephrotic-range proteinuria and oedema that manifest in utero or during the first 3 months of life^1^. In rare cases, CNS can be caused by congenital infections or maternal allo-immune disease, but most cases are caused by genetic defects in podocytes^2^. Several genes have been implicated in the aetiology of isolated CNS (most commonly *NPHS1*, which encodes nephrin^3,4^; *NPHS2*, which encodes podocin; *WT1*, which encodes Wilms tumour protein 1; and *PLCE1*, which encodes 1-phosphatidylinositol 4,5-bisphosphate phosphodiesterase ε1) or in less common syndromic forms of the disease (most commonly *WT1* or *LAMB2*, which encodes laminin subunit β-2)^5,6^. As pathogenic variants in these genes alter the physiology of podocytes, genetic forms of nephrotic syndrome are now referred to as podocytopathies^2^.

Patients with CNS are prone to severe complications such as haemodynamic instability, recurrent infections, thromboses and impaired growth. Most children with CNS progress to kidney failure within a few years^1,7–11^. In Finland, between 1965 and 1973, the mean survival of patients with CNS was reported to be 7.6 months (range 0–26 months) with most infants dying owing to infection or haemodynamic collapse^9^. In 1995, an aggressive treatment regimen including dialysis, early nephrectomy and transplantation was proposed and led to a dramatic improvement in survival^1^. With this regimen, more than 90% of patients with CNS could be transplanted with similar kidney and overall survival to other transplanted children^1,12^. Subsequently, numerous reports have emerged of successful treatment using a conservative approach involving optimized nutrition and medications without nephrectomy^13,14^.

In 2018, a joint initiative of the European Reference Network for Rare Kidney Diseases (ERKNet) and the European Society for Paediatric Nephrology (ESPN) established a Work Group to develop guidelines for the clinical diagnosis, management and treatment of CNS. As evidence regarding the optimal management of CNS is frequently missing or inadequate, here we provide a consensus report based on expert opinion rather than a clinical practice guideline. The genetic aspects of the hereditary forms of CNS are discussed further in a separate consensus statement^15^.

## Methods

We followed the RIGHT (Reporting Items for practice Guidelines in HealThcare) statement for practice guidelines^16^ and used the Delphi method. Three groups were assembled: a core leadership group, an external expert group and a voting panel. The core group comprised nine members of ERKNet and ESPN, including paediatric nephrologists and kidney geneticists, as well as a neonatologist, a kidney nurse and a patient representative (Supplementary Table 1). The external expert group included six paediatric nephrologists, an adult nephrologist, a kidney geneticist, a kidney pathologist, a paediatric pharmacologist, a neonatologist, a paediatric endocrinologist, an ethicist, a nurse and a patient representative, and the voting panel comprised 35 paediatric nephrologists from the ESPN nephrotic syndrome working group. The core group wrote the first draft of the consensus statement. Using an e-questionnaire, the members of the external expert group were asked to provide a level of agreement on the recommendations in the first draft using a five-point scale (strongly disagree, disagree, neither agree nor disagree, agree, strongly agree). Recommendations that did not reach a consensus of at least 70% were revised by the Core group. The revised draft was then sent to the voting panel. Recommendations that still did not reach a consensus of at least 70% were revised by the Core group and resent to the external experts and the process was repeated until a consensus level of at least 70% was achieved.

### PICO questions

We developed PICO (Patient or Population covered, Intervention, Comparator, Outcome) questions for this consensus statement^17^. The population was children with CNS (defined as onset of nephrotic syndrome within the first 3 months of life) before and after the initiation of dialysis or kidney transplantation. The intervention was treatment and the comparators were either no treatment or other treatment. The outcomes were recommendations for diagnosis, treatment and follow-up of children with CNS.

### Literature search

The following key words were used to identify relevant studies published before 31st December 2018: nephrotic syndrome, congenital nephrotic syndrome, Galloway Mowat Syndrome, Pierson Syndrome, Frasier Syndrome and Denys Drash Syndrome. The search retrieved 1,367 results but no randomized clinical trials; 54 articles are referenced in the consensus statement. Further details and a summary of the publications used for this consensus statement are included in Supplementary Table 2.

## Diagnosis

Infants with CNS present with nephrotic-range proteinuria and oedema with or without kidney failure. The diagnosis and management require expertise and a combined approach that can only be guaranteed in a centre with experience in treating this condition.

### Multidisciplinary management

We recommend that all patients with CNS are referred to specialized teams in tertiary paediatric nephrology centres and managed by multidisciplinary teams, including neonatologists, paediatric nephrologists, paediatric nephrology nurses, paediatric renal dieticians, paediatric surgeons, child and/or youth psychologists and social workers (Box 1). The psychosocial pressure that is often experienced by families with a child with CNS must be taken into account to enable successful management. All members of the multidisciplinary team must be trained in child care. When children with CNS are managed outside of a transplant facility, we recommend that they are introduced to a transplant centre early in the progression of their chronic kidney disease (CKD), with the aim of minimizing time on dialysis and facilitating the transplant process.

Box 1 Recommendations for diagnosis

- We recommend that all patients with CNS are managed by a multidisciplinary team.
- We recommend performing an initial diagnostic work-up, including medical history, clinical and biological evaluation of CNS complications and associated non-kidney features (Table 1).
- We recommend comprehensive genetic screening comprising all podocytopathy-related genes as a first-line diagnostic measure in every patient with CNS.
- We recommend providing genetic counselling promptly in families with a history of CNS or prenatal signs of CNS.
- We suggest that kidney biopsy be considered only in patients with sporadic, non-syndromic disease in whom comprehensive genetic testing has not yielded a molecular diagnosis.

### Initial diagnostic work-up

In infants with CNS, we recommend performing the initial diagnostic work-up as presented in Box 2. Extended diagnostic work-up aimed at identification of non-kidney manifestations of hereditary forms of CNS should also be considered (such as assessment of neurological status, sight, hearing, dysmorphic features and abnormal genitalia). The possible signs and symptoms of syndromic forms of CNS are discussed further in a separate consensus statement^15^.

Box 2 Initial work-up for a child with CNS **History**

- Family history: consanguinity, ethnicity, history of CNS, early infantile death and unsolved neurological and kidney diseases of infancy.
- Prenatal and perinatal history: enlarged prenatal nuchal translucency, increased amniotic fluid alpha-fetoprotein, fetal oedema, oligohydramnios and placental weight >25% of newborn weight.
- Patient history: fever episodes, pain, abdominal discomfort, swelling, fatigue.

**First-line evaluation**

- Growth chart: height or length, weight, head circumference if aged <2 years, calculation of BMI and annual height velocity.
- Blood pressure.
- Physical examination: volaemia, signs of oedema (e.g. ascites, pericardial and pleural effusions).
- Blood biochemistry: blood count, levels of sodium, chloride, albumin, magnesium, creatinine, urea, protein, albumin, cholesterol, fasting triglycerides and glucose.
- Levels of thyroid-stimulating hormone and free thyroxine (T4).
- Serum IgG level.
- Serum levels of ionized calcium, phosphate, alkaline phosphatase, PTH, 25(OH) vitamin D3.
- Ultrasound of abdomen and pleural space (kidney echogenicity and size, ascites, effusions and thrombosis).
- Cardiac ultrasound (effusions and left ventricular mass).

**Extended evaluation**

- Evaluation of dysmorphic features and skeletal abnormalities, genital examination, ophthalmological examination, hearing test.
- Full neurological examination and standardized assessment of cognitive status with or without brain MRI.
- Serology for syphilis, toxoplasmosis, CMV, rubella, measles, HBV, HCV, HSV1, HSV2, HZV, HIV and *Bordetella pertussis* (if the mother or infant has not already been screened for these infections).
- Further screening in selected patients in endemic areas or in the case of clinical suspicion: malaria, anti-nuclear antibodies, serum complement (C3 and C4), anti-neutral endopeptidase (NEP) antibodies, amino acids (for diagnosis of glutaric aciduria type I or sialic acid storage disease) and/or mercury levels).

**Genetic tests**

Refer to the ERKNet-ESPN consensus statement on genetic aspects of congenital nephrotic syndrome^14^.

**Genetic counselling**

As appropriate.

**Diet**

Assessment and advice from a renal dietician, including advice on salt, potassium, calorie and protein intake.

**Renal histology**

Kidney biopsy is indicated if all other screening is negative, indicating non-infectious, non-genetic CNS. Histological examination should include light microscopy, immunofluorescence and/or immunohistochemistry and electron microscopy.

### Genetic testing

Identification of a genetic cause of CNS establishes the aetiology, informs management, particularly with regard to the potential development of Wilms tumour or neurological involvement, and enables genetic counselling of the family. We therefore recommend genetic screening as a first-line diagnostic measure in every patient with CNS. The preferred method of genetic testing is massively parallel sequencing, with rapid whole-exome sequencing being the method of choice. In countries where rapid whole-exome sequencing is not yet clinically available, use of an extended podocytopathy gene panel is recommended owing to the wide phenotypic variability and genetic heterogeneity of the disease^4,5,18–21^. The minimum set of genes to be tested should include *NPHS1, NPHS2, WT1, PLCE1* and *LAMB2*. Screening of these genes identifies underlying genetic abnormalities in >80% of patients with CNS^4,5,18–20,22,23^. A dozen other less commonly mutated genes account for an additional ~5% of diagnoses. A clinical presentation that is suggestive of a particular syndromic form of CNS, such as Denys Drash Syndrome or Pierson Syndrome, or Finnish ethnicity, which is associated with founder pathogenic variants in *NPHS1*, may lead to direct testing of the suspected causative gene.

Disclosure of the results of genetic testing should involve recurrence risk counselling by a clinical geneticist or clinical counsellor. Families should be informed of the available options for prenatal testing as well as their risks to enable them to make an informed choice when considering whether to undergo such testing in subsequent pregnancies. Decisions regarding prenatal diagnostic testing, including pre-implantation diagnostics, should be discussed in light of the local financial, social and legal setting^24^.

The gene-specific management of CNS is detailed elsewhere^15^. Notably, children with the exonic *WT1* pathogenic variant must be monitored for Wilms tumour by performing abdominal ultrasound every 3 months until the age of 7 years^25^.

### Histopathology

As genetic screening identifies the underlying genetic abnormality in >85% of patients with CNS, non-invasive molecular diagnostic methods have largely replaced kidney biopsy in these patients^4,5,18–20,22,23^. We do not recommend routine kidney biopsy in patients with CNS. Kidney biopsy may be indicated in patients for whom a genetic diagnosis cannot be established or in those with a substantial reduction in eGFR (i.e. to <30 ml/min/1.73 m²) for whom a biopsy sample could be informative in establishing a rare diagnosis (such as congenital membranous nephropathy due to anti-neutral endopeptidase (NEP) antibodies or other glomerulopathies) and in estimating prognosis.

### Symptoms of mitochondrial disease

In patients with CNS, the following findings suggest an underlying mitochondrial disease: nystagmus, retinitis pigmentosa, visual impairment or loss, sensorineural deafness, developmental delay, cognitive impairment, hypotonia, seizure, encephalopathy, cardiomyopathy, feeding difficulties, liver failure, progressive muscle weakness, diabetes mellitus, lactic acidaemia, increased serum creatinine kinase, anaemia and/or pancytopenia. A few case reports exist of remarkable improvements in kidney function, but not in neurological sequelae, with coenzyme Q10 (CoQ10) supplementation in patients with CNS owing to mitochondrial disease^26,27^. We therefore suggest initiating a therapeutic trial of CoQ10 in patients with symptoms consistent with mitochondrial disease even before receiving the results of genetic testing. This therapy should be discontinued if no improvement in kidney function or substantial decrease in proteinuria is observed after 4–6 weeks^15^.

## Therapeutic management

**Fig. 1:**
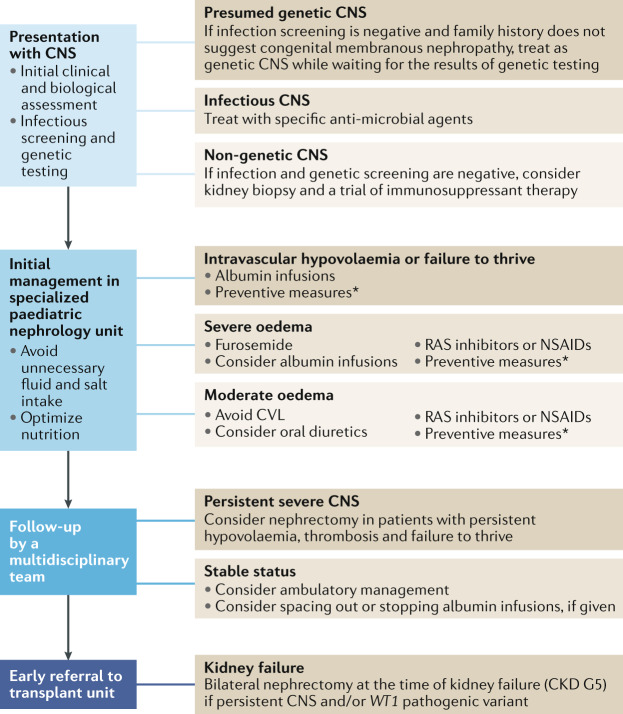
Opinion-based management algorithm for CNS. At presentation with congenital nephrotic syndrome (CNS), a clinical and biological assessment including screening for congenital infections and genetic analysis is recommended. Initial treatment should be based on the results of these assessments. Patients should be managed at diagnosis by a specialized paediatric nephrology team. Blood volume should be assessed and symptomatic treatments instituted to maintain blood volume and prevent complications. Follow-up must be managed by a multidisciplinary team. Nephrectomy can be considered for children with persistent, severe CNS despite optimal management. Stable children can be managed on an outpatient basis with spacing or even stopping of albumin infusions. All children should be referred promptly to a kidney transplant team. Bilateral nephrectomy is recommended at the time of kidney failure (chronic kidney disease (CKD) G5) if nephrotic syndrome persists and/or if the patient has a *WT1* pathogenic variant. *Preventive measures: prophylaxis for thrombosis, infection and anaemia, adequate nutrition and growth hormone substitution. RAS, renin–angiotensin system, CVL, central venous line.

CNS encompasses a wide spectrum of clinical phenotypes that should be managed with different approaches in specialized units. Some newborns and infants present with no or minimal symptoms and should not be given aggressive and potentially dangerous treatments, whereas others are critically ill with massive proteinuria, anasarca and haemodynamic compromise, and may require daily albumin infusions via a central venous line (CVL) and intensive symptomatic treatments to avoid complications. Management should therefore be adapted to the clinical severity of the condition with the aim of maintaining intravascular euvolaemia and adequate nutrition, as well as preventing complications (Box 3). As is typical for such a rare disease, considerable variability exists in clinical practice, with some centres aiming to avoid intensive treatment. As no conclusive clinical data, such as the results of randomized clinical trials, are available, we propose an opinion-based management algorithm for children with CNS (Fig. 1).

Box 3 Recommendations for fluid and albumin administration

- We recommend rapid referral of children with CNS to a specialized paediatric nephrology unit due to the complexity of the disease and fluid management.
- We recommend avoiding intravenous fluids and saline. Oral fluid intake should be concentrated if necessary to avoid marked oedema.
- We recommend using albumin infusions based on clinical indicators of hypovolaemia (including oliguria, acute kidney injury, prolonged capillary refill time, tachycardia, hypotension and abdominal discomfort) or upon failure to thrive. We do not recommend administering albumin infusions in children with CNS based on serum albumin levels.
- When possible, we recommend avoiding central venous lines in children with CNS owing to the high risk of thrombosis. If central venous access is required for repeated albumin infusions, we recommend administering prophylactic anticoagulation for as long as the line is in place (Box 7).

### General approach

We recommend rapid referral of children with CNS to a specialized paediatric nephrology unit. These children are often born prematurely and amniotic fluid may be stained with meconium but ventilator therapy is rarely needed. Pregnancy is usually uneventful. Given the wide variation in clinical findings in infants with CNS, individualized therapy is needed, with a number of key objectives: preserve all central and peripheral arteries and veins for potential dialysis access; avoid peripherally inserted catheters and unnecessary venepunctures^28^; optimize fluid, protein and caloric intake; minimize administration of salt-containing fluids; prevent thrombosis, particularly in patients with a CVL or hypovolaemia; and treat infection when clinically suspected by starting empiric antibiotics before culture results are available. C-reactive protein and leukocyte levels are not reliable indicators of septicaemia in patients with CNS^1^.

### Fluid management

As no studies have investigated specific treatments for oedema in CNS, treatment should focus on assessment of volume status (that is, overfill versus underfill) and salt restriction, as recommended for adult patients^29^. Fluid restriction is advocated for hyponatraemia and in the most severe cases of oedema.

Fluid prescription in patients with CNS should primarily be used to provide adequate nutrition. Intake of fluid should be limited, when feasible, by using concentrated high-calorie formulas to meet age-related energy needs, guided by the advice of expert renal dieticians. Intravenous albumin is the treatment of choice for acute symptomatic hypovolaemia (see below).

### Albumin infusions

The use of albumin infusions in children with CNS varies between centres. Some centres administer intravenous albumin only when deemed clinically indicated, whereas others use regular albumin infusion protocols (1–4 g/kg/day). Potential advantages of regular albumin infusions are replacement of lost protein to support growth and psychomotor development, stabilization of intravascular volume and minimization of oedema^1^. The disadvantages of regular albumin infusions are the need for a central line, which increases the risk of infection and/or thrombosis of large vessels, which endangers future haemodialysis access, the need for prolonged hospitalization (although home administration has been reported^30^) and the associated costs. Retrospective studies show no difference in long-term outcomes with these two strategies^13,14^. As most of the infused albumin is lost in the urine within hours, the purpose of albumin infusion is not to normalize serum albumin levels but to support intravascular volume and reduce extravascular fluid retention in patients with symptomatic hypovolaemia. Symptoms that are suggestive of hypovolaemia are prolonged capillary refill time, tachycardia, hypotension, oliguria and abdominal discomfort. Impacts on quality of life and school attendance should be taken into account when considering regular albumin infusions.

We acknowledge that some children with no or minimal symptoms do well without regular albumin infusions and do not need a CVL. Others may need frequent albumin infusions to prevent the clinical consequences of hypovolaemia and failure to thrive. In the latter, we recommend basing the frequency and dosage of albumin infusion on the clinical indicators of hypovolaemia listed above, rather than on serum albumin levels. In patients with severe disease, daily albumin infusions of up to 1–4 g/kg may be initiated. In stable patients or when CKD progresses, albumin dose may be reduced and infusions might subsequently be made less frequent or even stopped^13,14^.

### Vascular access

When possible, we recommend avoiding CVLs in children with CNS owing to the high risk of thrombosis and the need to preserve the vasculature for future haemodialysis access. However, when regular albumin infusions are inevitable, a CVL becomes necessary. In such cases we recommend administering prophylactic anticoagulation for as long as the line is in place (discussed further below). We also recommend avoiding peripherally inserted catheters and unnecessary venepunctures to preserve arteries and veins for the potential future creation of arteriovenous fistulae^28^.

### Diuretics

Diuretics should be used with caution and only in the case of intravascular fluid overload (as evidenced by good peripheral perfusion and high blood pressure), because they could induce or increase hypovolaemia and promote thrombosis (Box 4). In most children with CNS, diuretics improve oedema and fluid control and enable fluid administration to provide adequate nutrition, especially when given in conjunction with albumin infusions^10^. We recommend considering an intravenous bolus of furosemide (0.5–2 mg/kg) at the end of each albumin infusion^10,31^ in the absence of marked hypovolaemia and/or hyponatraemia.

In patients with severe oedema, we recommend commencing furosemide at 0.5–2 mg/kg per dose intravenously or orally up to six times daily (maximum 10 mg/kg per day) based on the degree of oedema and diuresis achieved. Adequate monitoring is required and should involve assessment of fluid status, electrolytes (the presence of hypokalaemia or hyponatraemia), blood pressure and kidney function (diuresis and estimated glomerular filtration rate). High doses of furosemide (>6 mg/kg/day) should not be given for periods longer than 1 week and infusions should be administered over 5–30 min to avoid hearing loss^32–34^. Furosemide must be stopped in the case of anuria.

In stable patients, furosemide can be given orally at doses of 2–5 mg/kg per day in combination with a thiazide or potassium-sparing diuretic with appropriate monitoring. Experimental evidence suggests that proteases in the urine, such as plasmin^35^, directly activate the epithelial sodium channel (ENaC) and thus contribute to salt retention and formation of oedema in patients with nephrotic syndrome^29,35,36^. As this direct activation of ENaC is independent of the mineralocorticoid receptor, it will not be inhibited by mineralocorticoid receptor blockers such as spironolactone. Therefore, if potassium-sparing diuretics are used, blockers of the ENaC, such as amiloride, are preferable to spironolactone.

Box 4 Recommendations for the use of diuretics

- If albumin infusions are given, we suggest administering a dose of furosemide (0.5–2 mg/kg) at the end of each infusion, unless the patient has marked hypovolaemia and/or hyponatraemia.
- We recommend using diuretics in patients with signs of intravascular fluid overload (as evidenced by good peripheral perfusion and high blood pressure in combination with oedema) and preserved kidney function.
- We recommend using furosemide (0.5–2 mg/kg per dose, intravenously or orally up to six times daily; maximum 10 mg/kg per day) dependent on the degree of oedema and achieved diuresis unless the patient has evidence of intravascular hypovolaemia. Dosages >6 mg/kg per day should not be given for periods longer than 1 week. We recommend administering infusions over 5–30 min to minimize ototoxicity.
- If a potassium-sparing diuretic is preferred, we recommend epithelial sodium channel (ENaC) inhibitors such as amiloride over mineralocorticoid inhibitors such as spironolactone.

### Anti-proteinuric agents

Renin–angiotensin–aldosterone system (RAAS) antagonists (angiotensin-converting enzyme (ACE) inhibitors or angiotensin type I receptor blockers (ARBs)) reduce glomerular protein loss via a dose-dependent haemodynamic effect (i.e. preferential dilatation of the efferent arteriole)^37^. In adults and older children with proteinuric nephropathies, a 30–50% reduction in proteinuria can typically be achieved with these drug classes^38^. In patients with CNS, the clinical effect of RAAS inhibition is usually moderate. A retrospective study reported that serum albumin levels were moderately increased (by a median of 6 g/l) and albumin infusion frequency was reduced in some, but not all, children with CNS who were treated with RAAS inhibitors^13^. When possible, RAAS inhibition should not be used before a post-term age of 4 weeks to avoid interfering with physiological RAAS functions in early postnatal tissue growth^39^ and/or long-lasting hypotension and oliguric acute kidney injury (AKI)^40^ (Box 5). The short-lasting ACE inhibitor captopril is preferred in young infants owing to its short half-life.

RAAS inhibition should be started at a very low dose and gradually increased with frequent monitoring of proteinuria, urine output, serum creatinine and potassium to the maximally effective and tolerated dose. The recommended captopril-dosing scheme for infants younger than 3 months is 0.01–0.5 mg/kg per dose, with a maximum daily dosage of 2 mg/kg. Older infants should receive 0.15–3 mg/kg per dose, with a maximum daily dosage of 6 mg/kg. If a therapeutic effect is observed, children who are in a stable condition may be switched to a long-acting ACE inhibitor (e.g. ramipril 0.1–0.2 mg/kg once daily) or ARB (e.g. candesartan 0.2–0.4 mg/kg once daily). There is no evidence to suggest that combined ACE inhibition and AT1 receptor blockade is more effective in reducing proteinuria than maximized ACE inhibitor or ARB monotherapy in children with CNS. We do not recommend use of such combinations owing to the increased risk of hypotension and AKI^41,42^.

Prostaglandin inhibitors (also called cyclooxygenase inhibitors) can reduce proteinuria by decreasing kidney perfusion and reducing intraglomerular pressure via suppression of renin production in the juxtaglomerular apparatus^43^. The efficacy of prostaglandin inhibitors in children with CNS is unclear because they are commonly administered in combination with other interventions such as ACE inhibitors, ARBs and/or unilateral nephrectomy. A retrospective study reported that combined treatment with an ACE inhibitor and the prostaglandin inhibitor indomethacin resulted in increased serum protein levels and sufficient growth and development in 4 of 5 children with CNS^44^. However, a subsequent study reported similar increases in serum albumin levels in 7 children who received combined ACE inhibitor and indomethacin therapy and 35 children who received ACE inhibitor monotherapy^13^.

To avoid adverse effects, such as oliguric AKI and erosive gastritis, non-selective prostaglandin inhibitors, such as indomethacin, should be started after the end of the neonatal period (post-term (adjusted) age >4 weeks) and dosed incrementally from 0.5 mg/kg/day to a maximum of 3 mg/kg/day. Prostaglandin inhibitors should be stopped in patients with advanced CKD (stage 4–5). Co-treatment with H2 blockers and/or proton pump inhibitors is recommended. Alternatively, selective prostaglandin G/H synthase 2 (also known as COX2) inhibitors such as celecoxib can be considered to minimize gastrointestinal adverse effects.

Treatment with diuretics, RAAS inhibitors and NSAIDs should be stopped in patients with or at risk of hypovolaemia (for example owing to gastrointestinal symptoms or worsening of hypoalbuminaemia) because they increase the risk of AKI and thrombosis in these patients^45^. Parents must be informed of the need to stop diuretics, RAAS inhibitors and NSAIDs if their child has vomiting or diarrhoea.

Box 5 Recommendations for antiproteinuric therapy

- We recommend administering RAAS-blocking therapy such as ACE inhibitors or ARBs in children with CNS aged >4 weeks.
- ACE inhibition should be started with the short-acting ACE inhibitor captopril, escalating the dosage from 0.01 to 0.5 mg/kg per dose in children younger than 3 months (maximum dosage of 2 mg/kg/day). Older infants should receive 0.15–3 mg/kg per dose (maximum dosage of 6 mg/kg/day).
- We do not recommend combining ACE inhibitors and ARBs owing to the potentially increased risk of acute kidney injury (AKI).
- In the case of poor responsiveness to RAAS blockade, we suggest considering the use of prostaglandin inhibitors as an add-on treatment (indomethacin dosed incrementally from 0.5 to 3 mg/kg/day).
- We recommend stopping prostaglandin inhibitors if no clinical benefit (that is, increase in serum albumin levels and/or reduction in oedema) is apparent after 2–4 weeks.
- In the case of non-kidney volume losses such as vomiting and diarrhoea, routine treatment with RAAS inhibitors, prostaglandin inhibitors and diuretics must be discontinued owing to the high risk of intravascular volume depletion and AKI.

### Nephrectomies

In a commonly used treatment protocol for CNS, bilateral nephrectomy is performed and dialysis initiated when the infant weighs around 7–9 kg (6–12 months of age) followed by kidney transplantation a few months later (upon attainment of 10 kg body weight). The mortality of these infants on dialysis is low (6–11%)^46,47^ and the risk of thrombotic events and septic infections is reduced after nephrectomy. However, many clinicians provide conservative therapy without nephrectomies and retrospective studies show no apparent difference in outcomes between these different treatment approaches^13,14^. An individualized, stepwise approach with prolonged conservative management is therefore an appropriate alternative to early bilateral nephrectomies and dialysis in many children with CNS (Box 6).

We suggest considering unilateral or bilateral nephrectomy in patients with severe complications including failure to thrive, thrombosis and/or difficulties in maintaining intravascular euvolaemia, despite optimization of conservative treatment. We recommend performing bilateral nephrectomies before kidney transplantation in patients with persisting nephrotic syndrome and/or a *WT1* dominant pathogenic variant.

Box 6 Recommendations for nephrectomies

- We do not recommend performing routine early nephrectomies in children with CNS.
- We suggest considering unilateral or bilateral nephrectomy in patients with severe complications, including failure to thrive, thrombosis and/or difficulty in maintaining intravascular euvolaemia despite optimization of conservative treatment.
- We recommend performing bilateral nephrectomies before kidney transplantation in patients with persisting nephrotic syndrome and/or a *WT1*-dominant pathogenic variant.

### Ambulatory management

When possible, we recommend ambulatory management of children with CNS to increase their quality of life, decrease their risk of nosocomial infections and reduce treatment costs. Patients with CNS are at risk of sudden deterioration, especially with acute infections. However, a retrospective study demonstrated no apparent difference in complications and long-term outcomes for patients treated in hospital or as outpatients^14^. Home administration of regular albumin infusions by parents following training by medical staff has been shown to be feasible and safe^30^.

### Treatment of non-genetic CNS

As CNS is most frequently caused by genetic abnormalities that are not targeted by immunosuppressive agents, we do not recommend using these agents to treat children with CNS (Box 7). Anecdotal reports exist of improvements in proteinuria in patients who received steroids and/or ciclosporin. However, these therapies were usually given in combination with ACE inhibitors, which likely explains the reduction in proteinuria^48–50^. Moreover, spontaneous remission has been reported in some patients with CNS^51^ and could potentially explain reports of seemingly beneficial effects of immunosuppressive agents. Negative genetic testing, negative infection screening results and a kidney biopsy sample excluding diffuse mesangial sclerosis should be obtained before considering immunosuppression^49,52^. If comprehensive genetic testing and screening for secondary forms of CNS yield negative results, a trial of immunosuppressive therapy may be considered.

Box 7 Recommendations for management of non-genetic CNS

- We do not recommend using immunosuppressive drugs to treat children with CNS.
- If comprehensive genetic testing and screening for secondary forms of CNS yield negative results, kidney biopsy and a trial of immunosuppressive therapy may be considered in selected cases.
- We suggest considering congenital membranous nephropathy owing to anti-NEP antibodies in patients who have kidney failure at presentation, transient proteinuria that resolves spontaneously or siblings with transient proteinuria at birth.
- We recommend treating patients with infection-related CNS with specific antimicrobial agents and performing genetic screening in these patients.

#### Congenital membranous nephropathy due to anti-NEP antibodies

A small number of infants presenting with a clinical picture of CNS may have congenital membranous nephropathy due to a maternal variant in the *MME* gene, which encodes the podocyte protein NEP. During pregnancy, the mother becomes sensitized by fetal NEP and produces anti-NEP antibodies that can damage the podocytes of the fetus, leading to nephrotic proteinuria^53,54^. The IgG anti-NEP titres become more elevated in subsequent pregnancies, resulting in a clinical picture ranging from either a miscarriage of the first pregnancy or no symptoms in the first child, to non-nephrotic transient proteinuria or severe CNS with kidney failure in subsequent children. Anti-NEP antibody^54,55^ screening should be carried out in patients with CNS who have kidney failure at presentation or transient proteinuria at birth that spontaneously resolves within a few weeks; those who have a family history of siblings with congenital membranous nephropathy or transient proteinuria at birth; and those who have membranous nephropathy on kidney biopsy (Box 7). In all of these cases the mother should also be tested for NEP antibodies. Patient management is mainly symptomatic^56^.

#### Infection-associated CNS

Patients with presumed infection-associated CNS may have an associated pathogenic gene variant and should be tested for underlying genetic causes of CNS^57^. We recommend treating infection-related CNS with specific antimicrobial agents (Box 7).

Congenital syphilis has re-emerged in many countries after years of declining incidence^58^. Kidney involvement in untreated newborns and infants can vary from mild proteinuria to nephrotic syndrome, haematuria or AKI, and might be the only presentation of the disease^59^. However, non-kidney features such as anaemia, jaundice, hepatosplenomegaly, cutaneous lesions and neurological symptoms are more frequent symptoms than kidney involvement. Diagnosis is based on the detection of antibodies against *Treponema pallidum* and *T. pallidum* haemagglutination assay. Treatment consists of penicillin G (50,000 U/kg intravenously every 12 h in patients aged ≤1 week, every 8 h in those aged >1 week and every 6 h in those aged >1 month) or benzathine penicillin G (50,000 U/kg, intramuscularly, every 24 h for 10–15 days)^58^.

Although >90% of congenital cytomegalovirus (CMV) infections are asymptomatic, CNS has been reported in infected patients. CMV-related CNS is extremely rare; more common presentations of CMV infection are convulsion, paraplegia, sensorineural hearing loss, absence of light reflexes and pulmonary and cutaneous lesions. Diagnosis is based on a positive PCR reaction showing the presence of viral DNA in urine and/or saliva. Treatment consists of ganciclovir (6 mg/kg, every 12 h for 15–21 days) followed by valganciclovir (15 mg/kg, every 12 h for 6 weeks). Of note, plasma concentrations of ganciclovir show large variations in newborns and the standard dose frequently fails to achieve the recommended target area under the concentration–time curve over 24 h (AUC_0–24_) of 40–50 µg*h/ml^60^. If possible, we suggest measuring ganciclovir AUC in cases of treatment failure and increasing the dose if the AUC is below this target.

Rare cases of infection-associated CNS have also been described in patients with congenital toxoplasmosis, hepatitis B and rubella^61^. Although HIV infection frequently causes nephropathy with proteinuria or nephrotic syndrome in children and adults, no patients with HIV-related CNS have been described to date.

## Complication monitoring and prevention

We recommend regular monitoring for and prevention of complications of CNS, including acute complications (such as hypovolaemic and hypervolaemic crisis, intermittent hypertensive and thromboembolic events, and bacterial and viral infections) and chronic consequences of the disease (including hypertension, dyslipidaemia, hypothyroidism, hypomagnesaemia, hypocalcaemia, vitamin D deficiency, bone disease, growth failure and progressive CKD) as well as adverse effects of medications and complications of prematurity (such as hyperbilirubinaemia) (Table 1, Box 8).

**Table 1:** Follow-up for a child with CNS

Assessment	Frequency during follow-up
***Clinical***	
Patient history (fever episodes, pain, abdominal discomfort, swelling, fatigue, adherence to medication)	At every visit
Physical examination including signs of oedema (e.g. ascites, pericardial and pleural effusions), tetany, skeletal status and extrarenal features	At every visit
Blood pressure	At every visit
Full neurological examination and standardized assessment of cognitive status	Monthly for 3 months, yearly thereafter
Growth chart: height or length, weight, head circumference if age <2 years, calculation of BMI and annual height velocity	Monthly for 3 months, every 3 months thereafter
***Biochemistry***	
Blood: complete blood cell count, sodium, chloride, ionized calcium, phosphate, magnesium, creatinine, urea, protein, albumin, cholesterol, fasting triglycerides and glucose	Monthly for 3 months, every 3 months thereafter or as appropriate
eGFR (Schwartz formula)	Every 3 months (more frequently in CKD stage 4)
ALP, PTH	Every 3 months, more frequently in advanced CKD (stages 4–5)
25(OH) vitamin D_3_	Every 6 months, yearly after age 12 months
TSH, free T4	Monthly for 3 months, thereafter every 3 months or as appropriate
IgG	Trough levels as appropriate
***Diet***	
Assessment and advice from a dietician including salt, K, calorie and protein intake	Monthly in infants, thereafter every 3 months
***Imaging***	
Ultrasound of abdomen and pleural space (kidney echogenicity and size, ascites, effusions, thrombosis)	Every 3 months until the age of 7 years in children with exonic *WT1* variant
X-ray of the left knee: mineralization and left wrist for bone age assessment in children aged >5 years	Yearly or as appropriate
***Extrarenal involvement***	
Assessment depending on the underlying disease	As appropriate
***Preparation for kidney replacement therapy***	
Referral to dialysis and/or transplant centre; preparation for dialysis including fistula creation and transplantation	Around 6 months of age and not later than when eGFR is <30 ml/min/1.73 m^2^

ALP, alkaline phosphatase; CKD, chronic kidney disease; eGFR, estimated glomerular filtration rate; PTH, parathyroid hormone; TSH, thyroid-stimulating hormone

Box 8 Recommendations for monitoring and prevention of complications **Thrombosis prophylaxis**

- We suggest that preventive anticoagulation should be considered in patients with CNS during states of increased thrombosis risk (owing to acute illness, risk of dehydration, inserted central lines and/or thrombocytosis >750,000/ml) and/or in patients with a previous thrombosis.

**Infection prophylaxis and management**

- We do not suggest routinely administering antibiotic prophylaxis in children with CNS; however, prompt antibiotic treatment should be started in the case of a suspected bacterial infection.
- We suggest that immunoglobulin infusions should be considered in patients with low serum IgG levels and recurrent or severe infections.
- We recommend following the vaccination schedule that is recommended for healthy children, including vaccinating against encapsulated bacteria and varicella-zoster virus (VZV), and administering the influenza vaccine annually.
- In the case of exposure to chickenpox in children who have not been immunized against VZV, we recommend prophylactic treatment with specific VZV intravenous immunoglobulins or oral acyclovir for 5–7 days starting within 7–10 days of the exposure.
- We recommend treatment of VZV infection with intravenous high-dose acyclovir for 7–10 days.

**Nutrition, growth and metabolism**

- We recommend provision of a diet with a high energy (130 kcal/kg/day) and protein (4 g/kg/day) content but low salt content (<0.5–3 g/day depending on the age of the patient).
- We recommend initiating growth hormone treatment in patients with persistent height growth failure despite adequate nutrition.
- We recommend supplementing with levothyroxine (T4) in the case of hypothyroidism.
- We recommend close monitoring of ionized calcium, 25-OH-D3 and PTH levels in children with CNS and supplementing with oral D3-vitamin (cholecalciferol) or 25-OH-D3-vitamin (calcifediol) and calcium (250–500 mg/day) in the case of low 25-OH-D3 and/or low ionized calcium and/or elevated PTH levels.
- We suggest considering use of statins when fasting LDL cholesterol is persistently elevated in patients with additional cardiovascular risk factors.

**Anaemia prevention and management**

- We recommend monitoring and treating iron deficiency and administering erythropoietin in patients who have anaemia despite iron supplementation.
- We recommend close monitoring of the reticulocyte count as a marker of erythropoiesis and response to therapy. Persistent anaemia after 4 weeks of iron and erythropoietin therapy requires further evaluation for other possible contributing factors, such as copper, ceruloplasmin or vitamin B12 deficiency, and appropriate treatment.

### Thrombosis prophylaxis

Patients with CNS are at risk of developing potentially life-threatening venous or arterial thromboembolic complications, including of the kidney, cerebral and/or pulmonary vessels^62^. In CNS, the thrombotic risk is multifactorial and includes a disease-related hypercoagulability, underlying thrombophilic predisposition and risks related to treatment (such as CVLs or diuretics). An inserted CVL is a strong pro-thrombotic risk factor in the nephrotic state and should be avoided whenever possible. In CNS, hypercoagulability is related to an imbalance between procoagulant and anticoagulant factors^63–65^. Urinary leakage of anticoagulant circulating factors (antithrombin III and plasminogen) and low molecular weight procoagulant factors (factor IX and factor XI) results in compensatory liver synthesis of high molecular weight procoagulant factors (fibrinogen, factor V, factor VII, factor VIII and factor X) resulting in hypercoagulability^66^. Moreover, patients with CNS are deficient in pituitary adenylate cyclase-activating polypeptide, which is a major inhibitor of megakaryopoiesis and of platelet activation, owing to urinary losses of pituitary adenylate cyclase-activating polypeptide bound to ceruloplasmin^67^. These findings theoretically justify the administration of platelet aggregation blockers in patients with CNS.

We suggest that preventive anticoagulation should be considered in all children with CNS and/or a prior thrombosis and during states of increased thrombosis risk (i.e. acute illness, risk of dehydration, inserted central lines and/or thrombocytosis >750,000/ml) (Box 8). The general goal of antithrombotic therapy is to prevent the formation, local extension and recurrence of thrombosis, embolism, and long-term complications. Infusion of antithrombin III (ATIII; 50 units/kg) before the placement of a central venous catheter is recommended^10^. Agents that have been used for antithrombotic prophylaxis in nephrotic syndrome include heparin, vitamin K antagonist and aspirin^64,65^. Anticoagulation with low molecular weight heparins may be ineffective owing to reduced antithrombin III levels. In Finnish patients with CNS and CVLs, long-term warfarin prophylaxis (target international normalized ratio 2–2.5) has been routinely used for decades and no substantial increase in the risk of bleeding has been observed (T. Hölttä, unpublished data)^68^. However, the clinical benefit of warfarin has not been substantiated in a controlled trial. Although early reports suggest that anticoagulation might prevent cerebral thromboses in children with CNS^1^, a recent retrospective outcome study reported that anti-thrombotic prophylaxis with warfarin, heparin or aspirin did not change the incidence of thrombotic events^13^. As aspirin might induce AKI^69^, when considered for patients with CNS it should be used with caution and high doses should be avoided. Case reports exist of successful use of direct-acting oral anticoagulants that inhibit factor Xa as thromboprophylaxis in adults with nephrotic syndrome^70^. Magnesium and calcium supplements should be given to children with CNS when necessary to avoid very low levels that may promote thromboses^71^.

### Infection prophylaxis and management

#### Antibiotics

Infections are a major concern and the primary cause of death in children with CNS^1,10,13,14^. These children are prone to infections caused by encapsulated bacteria such as pneumococci because of urinary losses of IgG and complement opsonins. However, prophylactic antibiotics are not routinely indicated as several studies have shown that they are not associated with a significant reduction in the rate of sepsis^1,11,14,72^. Appropriate therapeutic antibiotics should be started promptly in patients with proven or suspected acute bacterial infection^1^ (Box 8).

#### Immunoglobulin infusions

As mentioned above, patients with CNS can have extremely low levels of circulating IgG owing to urinary losses. However, the use of prophylactic intravenous immunoglobulins (IVIGs) is much debated. Arguments against systematic infusions include rapid urinary loss (up to 50% of infused IgG is lost in 30 h)^73,74^; the fact that commercial immunoglobulin preparations contain low titres of IgG against the bacteria that are most commonly responsible for septic episodes (staphylococci, streptococci and Gram-negative bacteria)^1,10^ and the high cost of immunoglobulin preparations.

IVIG in combination with parenteral antibiotics may be useful to treat septic episodes in children with low plasma IgG levels^10^. Preventive IVIG infusions may also be considered in the case of low plasma total IgG levels and recurrent and/or severe infections, similar to the management of secondary hypogammaglobulinaemia owing to causes other than CNS^75^.

#### Vaccination

Vaccination should follow the recommended schedule for healthy children, including vaccinating against encapsulated bacteria (especially meningococcal, *Haemophilus influenzae* and pneumococcal) and varicella-zoster virus (VZV)^76,77^. We also recommend annual vaccination against influenza.

#### Prevention and treatment of VZV infection

In the case of exposure to chickenpox, we recommend treating susceptible patients (i.e. those with hypogammaglobulinaemia who are not immunized against VZV and do not have a history of chickenpox) with VZV immunoglobulins (VZIGs) as soon as possible (Box 8). This strategy may be effective for reducing the severity of chickenpox symptoms when VZIGs are given up to 10 days after exposure^78^. If VZIGs are not available, we recommend prophylactic treatment with oral acyclovir (10 mg/kg four times a day for 7 days) within 7–10 days of exposure to chickenpox^68,79,80^.

Diagnosis of VZV infection relies on clinical features with or without the use of PCR to detect the virus in vesicle samples from the skin. Of note, specific antibody titres are not informative in children with CNS who have nephrotic-range proteinuria and are unreliable in those who are receiving IVIG infusions. We recommend treatment of VZV infection with intravenous high-dose acyclovir for 7–10 days.

### Nutrition, growth and metabolism

We recommend a diet with high energy (130 kcal/kg per day) and protein content (4 g/kg per day) but low salt content (<0.5 g per day in babies aged <6 months, <1 g per day in infants aged 7–12 months, <2 g per day in children aged 1–3 years and <3 g/day in children aged >3 years)^81^ (Box 8). Patients should be followed by an expert dietician and enteral tube feeding or gastrostomy should be considered in those with insufficient oral intake. Fluid restriction should not compromise caloric intake.

There is no evidence that pervasive growth hormone deficiency and growth failure in CNS is likely related to nutritional deficiencies and CKD. If nutritional deficiencies have been excluded, growth hormone (0.045–0.05 mg/kg/day subcutaneously) may be administered from the age of 6 months in children whose height is <3rd percentile, height velocity is <25th percentile and eGFR is ≤60 ml/min/1.73 m^2^ (ref.^82^). As a persistently reduced growth rate ultimately results in short stature, growth hormone therapy may also be considered in children with height below the 10th percentile who have a low height velocity (<25th percentile) that persists beyond 3 months in infants and beyond 6 months in children with growth potential, provided that other potentially treatable risk factors for growth failure such as malnutrition or metabolic acidosis have been adequately addressed^82^.

Hypothyroidism in CNS occurs as a result of urinary loss of thyroxine-binding proteins. We recommend measuring free thyroxine and thyroid-stimulating hormone (TSH) at disease onset and treating hypothyroidism as indicated by laboratory testing^1^.

Children with nephrotic syndrome have low 25-hydroxyvitamin D3 (25-OH-D3) levels owing to urinary loss of vitamin D-binding protein. As total serum calcium levels underestimate calcium content in the presence of hypoalbuminaemia, estimation of vitamin D deficiency is not accurate in these children. We recommend close monitoring of ionized calcium, 25-OH-D3 and parathyroid hormone (PTH) levels in children with CNS and supplementing with oral D3 vitamin (cholecalciferol) or 25-OH-D3 vitamin (calcifediol) and calcium (250–500 mg/day) in those with low 25-OH-D3 and/or low ionized calcium and/or elevated PTH levels. Reduced levels of ionized calcium and elevated PTH levels indicate the need for vitamin D and calcium supplementation^10^. Vitamin D supplementation has been shown to correct vitamin D deficiency in children with nephrotic syndrome^83^. We suggest considering use of statins when fasting LDL cholesterol is persistently >160 mg/dl (4.1 mmol/l)^84,85^ or >130 mg/dl (3.4 mmol/l) in patients with additional cardiovascular risk factors such as hypertension and obesity^86^.

### Anaemia prevention and management

Successful correction of anaemia in patients with nephrotic syndrome depends on the underlying cause, which may be one of or a combination of the following: urinary losses of erythropoietin (EPO), iron, transcobalamin and transferrin (transferrin saturation and ferritin level are unreliable in CNS); vitamin B12 and/or copper deficiency; and ACE inhibitor toxicity^87^. Iron deficiency anaemia should be treated with iron supplementation. As massive urinary losses of EPO are expected in CNS, a trial of EPO therapy should be considered in patients with anaemia after correction of iron deficiency. Recombinant human EPO has been reported to be safe and efficacious for the treatment of anaemia in children with nephrotic syndrome^87,88^. Increased doses of EPO are often required owing to urinary losses^87^ and subcutaneous administration of EPO might be superior to IV administration. We recommend close monitoring of the reticulocyte count as a marker of erythropoiesis and response to therapy (Box 8). Persistent anaemia after 4 weeks of iron and EPO therapy requires further evaluation for other possible contributing factors, such as copper, ceruloplasmin or vitamin B12 deficiency, followed by appropriate treatment.

## Management of kidney failure

A retrospective case note review by members of the ESPN Dialysis Working Group reported that infants with CNS who require dialysis have rates of peritonitis, dialysis technique survival, growth and transplantation that are comparable with those of infants with other primary kidney diseases^46^. Peritoneal dialysis is the modality of choice for children with CNS because it preserves central venous access; however, haemodialysis is an alternative with comparable outcomes (Box 9). In patients with autosomal recessive disease, parental kidney donation is usually accepted.^15^

Mild proteinuria after kidney transplantation is not rare and can be related to several conditions including graft rejection, recurrence of primary glomerulopathy, de novo glomerulopathy, infection or drug toxicity^89^. Recurrence of nephrotic-range proteinuria has also been described in patients with CNS after kidney transplantation^90–92^. Almost all children with CNS who have recurrence of nephrotic range proteinuria after kidney transplantation have a homozygous *NPHS1* p.Leu41Aspfs variant (known as Fin-major) that leads to an early stop codon and total absence of nephrin in the native kidney. Post-transplant de novo glomerulopathy occurs in 25–35% of these patients and at least 70% of those with post-transplant glomerulopathy have detectable anti-nephrin antibodies caused by allo-immunization against the nephrin molecule in the kidney graft. Recurrence can occur at any time after transplantation, kidney function is initially normal despite heavy proteinuria, and kidney biopsy samples show only mild histological changes with negative immunofluorescence^89,93–95^. Recurrence of nephrotic-range proteinuria in children of other genetic backgrounds is very rare and only one patient with anti-nephrin antibodies has been reported outside Finland; this patient carried a homozygous *NPHS*1 truncating variant (p.Glu189Ter*)*^90^. Successful treatment outcomes have been reported after treatment with daily plasma exchanges, methylprednisolone pulses and oral cyclophosphamide or rituximab^90,93^.

Early or late recurrence of nephrotic range proteinuria has also been reported in 1–2% of patients with homozygous or compound heterozygous pathogenic variants in the podocin gene (*NPHS2*, especially p.Arg138Ter and p.Arg138Gln variants)^91^. The pathophysiology of post-transplant de novo glomerulopathy in patients with *NPHS2* pathogenic variants is unclear (causative antibodies have not been identified) and might be multifactorial^92^.

Box 9 Recommendations for kidney failure

- We recommend that use of dialysis in children with CNS follows the general guidelines for kidney replacement therapy in infants and children.
- In children with post-transplant proteinuria, we recommend considering antibody-mediated disease and antibody reduction strategies (i.e. plasmapheresis and immunosuppressive drugs).

## Primary outcome measures

Patients with CNS are prone to developing severe complications, including growth failure, cognitive delay, thromboses, hypothyroidism, infections, hypertension and anaemia, which may require frequent hospitalizations and considerably impair their quality of life. We recommend aiming for normal growth, nutritional status and cognitive and motor development; preservation of vascular access (patent central veins and peripheral vessels for fistulae); absence of thrombotic complications, severe infections, oedema and anaemia; normal blood pressure; euthyroidism; minimized hospitalizations and good quality of life (that is, absence of pain and the ability to perform normal age-appropriate daily activities) in these patients. These goals should be regularly monitored as primary outcome measures.

## Ethical considerations

A number of ethical issues should be considered when taking care of a child with CNS. Decisions about intensive versus palliative treatments in neonates with severe and life-threatening disease should be made by a team of professionals in a family-centred shared decision-making framework led by the primary responsible physician^96,97^. In patients with severe comorbidities and/or under circumstances with limited medical resources, the decision to withhold treatment can be taken by the medical team after discussion with the family.

Specific literature on offering genetic testing to siblings of children with autosomal dominant CNS (which results in phenotypic heterogeneity in disease expression) is lacking. In general, genetic counselling of the family should precede genetic testing^98^. Genetic testing of asymptomatic siblings for the known pathogenic causative variant in a patient with CNS should be considered only in the case of WT1-associated glomerulopathy as this is the only autosomal dominant disorder with variable expressivity and incomplete penetrance that can manifest as CNS^99^.

## Future research

We recognize the paucity of scientific evidence in the field of CNS. Indeed, during our literature search we identified 54 relevant articles but no randomized controlled trials. Compelling clinical questions remain unanswered and we propose a number of research themes to address these questions (Box 10).

Box 10 Future research

- Develop a comprehensive registry for children with CNS to evaluate the variations in treatment and natural history of the disease, including rare complications.
- Evaluate the impact of CNS on schooling, social life and professional activity.
- Evaluate phenotype–genotype correlations in CNS.
- Define the optimal indications, dose and frequency of albumin infusions to use once patients have achieved a stable disease state.
- Evaluate the risk versus benefit ratio of approaches to preventing and/or treating CNS complications, such as use of anticoagulation, immunoglobulin infusion and vaccinations.

## Conclusions

In these recommendations, we provide guidance to multidisciplinary teams for the initial diagnostic work-up and monitoring of complications in children with CNS. We recommend prompt genetic screening in all children with CNS and genetic counselling of their families. Routine kidney biopsy is not recommended but may be considered in patients with sporadic, non-syndromic disease if comprehensive genetic testing has not yielded a molecular diagnosis. Therapeutic management should be adapted to the clinical severity of the condition with the aim of maintaining intravascular euvolaemia and adequate nutrition, preventing complications such as infections, thrombosis, psychomotor delay and failure to thrive, and preserving the vasculature. We recommend basing the use of albumin infusions on clinical indicators of hypovolaemia or on failure to thrive, rather than on serum albumin levels. When possible, we recommend avoiding CVLs owing to the high risk of thrombosis. We provide guidance for symptomatic treatment of CNS, including use of ACE inhibitors or ARBs, diuretics, anticoagulation during states of increased thrombosis, vaccination and IVIG infusions in selected patients. We do not recommend performing routine early nephrectomies but suggest that they are considered in patients with severe complications, despite optimization of conservative treatment, and before transplantation in patients with persisting nephrotic syndrome and/or *WT1*-dominant pathogenic variants.

## Supplementary information

Supplementary Information

## Glossary

Podocytes: Highly specialized glomerular epithelial cells that are the main components of the glomerular filter.
Megakaryopoiesis: The process by which megakaryocytes develop from haematopoietic stem cells.
