# Supplementary material for: Management of congenital nephrotic syndrome: consensus recommendations of the ERKNet-ESPN Working Group
Source: Nat Rev Nephrol. 2021 Jan 29;17(4):277–89. doi: 10.1038/s41581-020-00384-1 (PMC8128706; doi:10.1038/s41581-020-00384-1)
Supplement: Supplementary file 1 — Supplementary Information [file 41581_2020_384_MOESM1_ESM.pdf]

nature portfolio

https://doi.org/10.1038/s41581-020-00384-1

Supplementary information

Management of congenital nephrotic syndrome: consensus recommendations of the ERKNet-ESPN Working Group

In the format provided by the authors and unedited

Management of Congenital Nephrotic Syndrome: Consensus Recommendations by the ERKNet-ESPN Working Group

Olivia Boyer, Franz Schaefer, Dieter Haffner, Detlef Bockenhauer, Tuula Hölttä, Sandra Bérody, Hazel Webb[6] Marie Heselden, Beata S Lipska-Ziętkiewicz, Fatih Ozaltin, Elena Levtchenko and Marina Vivarelli

SUPPLEMENTAL MATERIAL

Supplementary Table 1: Area or expertise and responsibilities of core group members

Supplementary Table 2: Evidence review

Supplementary Table 1: Area or expertise and responsibilities of core group members

Name	Area of expertise	Responsibilities
Bérody, Sandra	Neonatology	Neonatal management<br>Management of complications
Bockenhauer, Detlef	Pediatric Nephrology<br>Nephropathology<br>Guideline development	Edema control, indications for nephrectomy
Boyer, Olivia	Pediatric nephrology<br>Guideline development	Guideline development<br>Management of complications, dialysis
Haffner, Dieter	Pediatric nephrology<br>Guideline development	Methodology, prostaglandin inhibitors, diuretics, outcome measures
Hölttä, Tuula	Pediatric nephrology	Post-transplant recurrence
Levtchenko, Elena	Pediatric nephrology<br>Guideline development	Management of complications, of infectious and immune causes, ethics
Lipska-Ziętkiewicz, Beata S.	Clinical Genetics,<br>Genetic Counselling,<br>Renal genetics	Genetic diagnostics and management
Ozaltin, Fatih	Pediatric nephrology<br>Renal genetics	Genetic diagnostics and management
Schaefer, Franz	Pediatric nephrology<br>Guideline development	RAS inhibition, prostaglandin inhibition, outcome measures
Vivarelli, Marina	Pediatric nephrology<br>Guideline development	Diagnostics and management, prevention of complications, indications for biopsy
Webb, Hazel	Pediatric nephrology nursing	Management of complications<br>Ambulatory management
Heselden, Marie	Patient representative	

Supplementary Table 2 : Evidence review

No	1st author, year, country of origin [Ref.]	Title of Publication	Study design	Key-words	Patients	Intervention and comparator	Outcomes
**Interventions (Observation Studies)**							
1	Bérody S. [1]<br><br>Nephrol Dial Transplant<br><br>(2018)<br><br>France<br><br>PMID: 29474669<br>DOI: 10.1093/ndt/gfy015	**Treatment and Outcome of Congenital Nephrotic Syndrome**	Nationwide retrospective study on 55 consecutive children born between 2000 and 2014 treated for non-infectious CNS.<br><br>18 French pediatric nephrology centers	Albumin<br><br>Chronic renal failure<br><br>Nephrotic syndrome<br><br>Quality of life<br><br>Survival analysis	N=55 analyzed from 51 families (46% consanguinity)<br><br>Sex (F/M): 0.9:1<br><br>Median Follow-up: 44 month (1-168)<br><br>N=17/55 relative with congenital, infantile or steroid-resistant NS<br><br>N=6/55 history of intrauterine fetal death in a sibling	**Daily albumin Infusion from diagnosis:**<br>N=53/55 (96%);median age 14 days<br>Target albumin level:>20g/L<br>Mean dosage: 2.5 6 4.3 to 3.4 6 2.4 g/kgBW<br><br>N=10/53 (18%) albumin withdrawn with normal eGFR; Median age 11 month (5-29)<br>*N=7/10 `mild' NPHS1 variant*<br>*N=1/10 homozygous `severe' NPHS1 variant*<br>*N=1/10 compound heterozygous NPHS2*<br>*N=1/10 heterozygous NPHS1 (considered as not mutated)*<br>*N=2/10 ESKD (12 / 54 mo of age)*<br>*N=8/10 remained free from albumin substitution at last follow-up*<br><br>**Substitution of i.v. Immunglobulins (IVIg)**:<br>N=24/55 (43%)<br><br>**Prophylactic Phenoxymethylpenicillin:**<br>N=22/55 (40%)<br><br>**ACEi:** N=48/55 (87%)<br><br>**Indomethacin:** N=28/51(45%)<br><br>**Anticoagulation:** N=51/55 (92%)	**Estimated cumulative incidence**<br>0.5 per 100,000<br><br>**Underlying genetic defect:**<br>Biallelic *NPHS1*: N=37/55 (65%); 7 novel variants<br>Biallelic *NPHS2*: N=5/55 (7%)<br>Heterozygous WT1: N=4/55 (7%)<br>Not identified: N=9/55 (16%); N=2/9 no DNA<br>*N=2/19 regularly outpatient clinic*<br>N=35/54 hospitalization until dialysis or death<br><br>**Infectious and Thrombotic Complications**<br>Infection rate: 2.41/patient/year<br>N=39/55 (71%) total 120 bacterial infections (0-8/patient); n.s. compared between different groups<br>Catheter sepsis rate: 1.82/patient/year; n.s. compared between different groups<br><br>22 reported thrombotic episodes in N=16/55 (40%); catheter thrombosis 0.25/patient<br>*N=2/16 (13%) cerebrovascular accidents*<br><br>**Kidney and Patient Survival**<br>N=39/55 (71%) ESKD median age 11 mo (0-63)<br>*N=19/39 (49%) pre-emptive bilateral nephrectomy, median age 10 mo (2-27=*<br>*N=19/39 (49) natural course of disease, median age 13 mo (0-63)*<br>*N=1 N/A*<br><br>Median age of ESKD significantly higher in children with initial creatininemia <50µmol/L compared to children >50µmol/L (12 mo vs. 2 mo, p<0.005)

							Renal survival:<br>Year 1: 65%<br>Year 2: 45%<br>Year 3: 21%<br>Year 5: 5%<br><br>Dialysis:<br>N= 33/55 (60%) median age 11 mo (1-54)<br>*N=7/33 (21%) died on dialysis*<br><br>Transplantation:<br>N=24/55 (44%) median age 29.5 mo (18-65); no post-transplant recurrence of NS<br><br>Death:<br>N=13/55 (24%)<br>*N=4/13 at diagnosis*<br>*N=2/13 from ESKD at 1 mo of age*<br>*N=1/13 heart arrhythmia after PD*<br>*N=2/13 nosocomial septic shock*<br>*N=6/13 on dialysis after bilateral nephrectomy (various causes)*<br>*N=1/13 sudden death at 5yr old transplanted child from heart failure*<br><br>**Outcome of children with *NPHS1* variant vs other variants**<br>N=25/37 (68%) reached ESKD vs N=14/18 (78%) with no or other variant; p=0.53<br>Median age (mo) of ESKD:13 (6-54) vs 3.5 (0-64); **p<0.005**<br>Median age (mo) at death: 13.5 (7-64) vs 4(1-16), **p<0.02**<br><br>No phenotype-genotype correlation between 'mild' and 'severe' *NPHS1* variants.
2	Dufek S. [2]<br><br>Nephrol Dial Transplant<br><br>(2018)<br><br>UK	**Management of Children with Congenital Nephrotic Syndrome: challenging treatment paradigm**	Retrospective 6-year review 1 Jan 2010-31 Dec 2015 members of ESPN Dialysis Working Group	bilateral nephrectomies,<br><br>CNS,<br><br>genotype–phenotype correlation,	N=80 (from 17 tertiary nephrology units)<br><br>Sex (M/F): 40/40<br><br>90% Caucasian<br>10% Asian<br>6% Arabic	**Albumin Infusion:**<br>N=68/71 (96%); median age of 9 days (3-47)<br>S-Albumin 10 (8-13) g/L<br>Median dose 2 (1-3) g/kg/dose and 7 (4-7) sessions/week<br><br>N=14 (20%) received albumin infusions at home	**Genetics:**<br>*NPHS1*: N=55 (69%)<br>*WT1*: N=9 (11%)<br>*NPHS2*: N=1 (1.3%)<br>*LAMB2*: N=2 (2.5%)<br>*PLCE1*: N=1 (1.3%)<br>*SGPL1*: N=1 (1.3%) **Novel gene variant**<br><br>No causative variant: N=11 (14%)

	PMID:30215773<br>DOI: 10.1093/ndt/gfy165		22 centers in 15 countries	management<br><br>NPHS1		**ACEi:**<br>N=42/71 (59%) median age of 57 days (28-81)<br>*N=7/42 (17%) ACEi + indomethacin*<br><br>S-Albumin pre- and post ACEi available only in 33/71(79%) children<br><br>**Anti-thrombotic therapy:**<br>N=45/71 (63%)<br><br>**Nephrectomy:**<br>N=33/71 (47%)<br>*N=29/33 (87%) NPHS2*<br>*N=4/33 (12%) without confirmed genetic cause*<br><br>N=4/33 (12%) unilateral (age: 6 mo (4-11)<br>N=6/33 (18%) bilateral two steps<br>N=23/33 (70%) bilateral one step (age: 9 mo (7-16))	*N=3 (3.8%) DMS*<br>*N=2 (2.5%) FSGS*<br>*N=1 Galloway Mowat & Pierson*<br>*N=2 uncertain / nonpathogenic variant in Nphs1 / WT1*<br><br>**ACEi treatment**<br>No difference between children only on ACEi compared to ACEi+indomethacin (p=0.3).<br><br>Increase in S-Albumin:<br>N=14/21 (67%) NPHS1<br>N=1/1 (100%) NPHS2<br>N=8/11 (78%) all others<br><br>**Anti-thrombotic therapy:**<br>N=9/71 (13%) developed thrombosis<br>*N=4/26 (15%) not on prophylaxis*<br>*N=5/45 (11%) on prophylaxis (warfarin n=3, heparin n=1 ,aspirin n=1)→ p=0.06*<br><br>**Comparison bilateral nephrectomy vs. conservative treatment (only *NPHS1* variant N=55) -**N=13/55 excluded-<br>N=25 nephrectomized<br>N=15 conservative treatment<br>Median follow up from birth:<br>35 (22-40) month vs 33 (22-54) month<br><br>ACEis: 28% vs. 94%, p<0.001<br>Dialysis: 100% vs 35% , p<0.001<br>ESKD: 8 month vs 25 month, p<0.001<br>Median renal survival: 8 (CI 6-10) vs 45 (CI 26-64) month<br>Transplantation (N=24): 80% vs 24%, p<0.001<br>Tx age: 17 vs 33 month, p<0=0.005)<br><br>Complications<br>Peritonitis: 32% vs 13%, p=0.16<br>Central line infection: 48% vs 47%, p=0.95<br>Septic episodes: 54% vs 53%, p=0.94<br>Thrombotic events: 16% vs 12%, p=0.70

							Recurrence of NS post Tx: 3/15 (12%) vs 1/25 (4%)<br><br>Death due to cardiovascular cause:<br>N=1/25 (4%) at 13 month of age (NX group)<br>N=2/17 (12%) at 33 and 49 mo of age (conservative group)<br><br>**Phenotype-Genotype Correlation in *NPHS1* variant and conservative treatment (N=17)**<br><br>In the conservative group, 8 of 17 children had variants classified as 'severe' and 9 had at least one variant classified as 'milder'. Baseline parameters were comparable : gestational age; birthweight; age at presentation; S-albumin and S-creatinine at presentation. A comparable percentage of children in both groups showed an increase in S-albumin with ACEi therapy (75% versus 80%; P.0.86). Renal survival and age at start of dialysis and patient survival
3	Dufek S. [3]<br><br>Pediatr Nephrol (2018)<br><br>UK<br><br>https://doi.org/10.1007/s00467-018-4122-0	**Infants with congenital nephrotic syndrome have a comparable outcome to infants with other renal diseases**	Retrospective 6-year review 1 Jan 2010-31 Dec 2015 members of ESPN Dialysis Working Group<br><br>22 centers in 15 countries	CNS<br><br>Infant<br><br>Dialysis<br><br>Complication	N=80 (from 17 tertiary nephrology units)<br><br>Sex (M/F): 40/40<br><br>90% Caucasian<br>10% Asian<br>6% Arabic	**Dialysis:**<br>N=55/80 (69%)<br>*N=34/55 (62%) NPHS1*<br>*N=1/1 (100%) NPHS2*<br>*N=9/9 (100%) WT1*<br>*N=11/15 (73%) other genetic causes*<br><br>**Short Term Dialysis <3 months:**<br>N=11/55 (14%)<br><br>**Chronic Dialysis ≥3 months:**<br>N=44/55 (55%)<br>*N=25/44 (57%) NPHS1*<br>*N=1/44 (2%) NPHS2*<br>*N=7/44 (16%) WT1*<br>*N=11/44 (25%) other diagnosis*<br><br>Median age at start: 8 mo (4-14)<br><br>Dialysis required:<br>N=17/44 (39%) by age 6 mo<br>N=30/44 (69%) by 1 yr<br>N=40/44 (91%) by 2 yrs	**Median renal survival:**<br>NPHS1: 15 month (95% CI 8.6-21.4)<br>NPHS2: 30 month (95% CI ?)<br>*WT1*: 2 mo (95% CI 0.6-3.4)<br>All others: 10 mo (95% CI 0.3-19.7)<br><br>**Complications on PD:**<br>N=20/41 (49%) peritonitis<br>Peritonitis rate 0.77 per year at risk<br><br>No difference in patients with or without nephrectomy<br>N=0/41 (0%) thrombosis<br><br>N=10/41(24%) hernias<br><br>N=3/41 (7%) pleural effusion (2 switched to HD)<br><br>**Complications on HD:**<br>N=1/17 (6%) CVL infection<br>N=0/17 (0%) septic episodes

						**<u>PD:</u>**<br>N=41/44 (91%) PD<br>N=13/41 (31%) CCPD with day exchange or dry day<br>N=8/44 (20%) CCPD with last bag fill in<br>N=6/44 (15%) ambulatory PD<br>N=1/44 (2%) tital PD<br><br>N=14/41 (34%) switch to HD<br><br>**<u>HD:</u>**<br>N=3/44 (7%), no switch to PD<br><br>Children started are PD younger than those with HD: 7 vs 17 month, **p=0.05**	**<u>Final Follow-up:</u>**<br>Median age 31 (14-49) month<br><br>N=29/80 (36%) transplanted patients the during study,<br><br><u>median age at transplantation:</u> 22(14-28) mo<br><br><u>median time on chronic dialysis before transplantation:</u> 9 (6-18) mo<br><br><u>death:</u> N=14/80 (18%)<br>*N=3/14 (21%) within 3 months of dialysis*<br>*N=5/14 (36%) on chronic dialysis (4 PD; 1HD); median age of death 11(5-51) mo*<br><br><u>Causes of death on dialysis:</u><br><u>N=3/14 (21%) sepsis (4PD, 1HD)</u><br><u>N=2/14 (12%) cardiac arrest (1PD, 1HD)</u><br><u>N=1/14 (7%) severe prematurity (PD)</u><br><u>N=2/14 (14%) withdrawal of active treatment (1 PD, 1 CVVH)</u>
4	Hölttä T.[4]<br><br><u>Pediatr Nephrol</u><br><br><u>(2016)</u><br><br><u>Finland</u><br><br>https://doi.org/10.1007/s00467-016-3517-z	**Timing of renal replacement therapy does not influence survival and growth in children with congenital nephrotic syndrome caused by variants in NPHS1: data from the ESPN/ERA-EDTA Registry**	Retrospective study<br><br>Data from Jan 1991 – 31 Dec 2012 collected<br><br>Within ERA-EDTA/ESPN registry	CNS<br><br>*NPHS1*<br><br>Kidney<br><br>Transplantation<br><br>Graft Survival<br><br>Pediatrics	Total N=170 CNS patients<br><br>100% *NPHS1* variants<br><br>initiated RRT<br><br>Sex M/F: 79/91<br><br><br>**Finnish patients:**<br>N=66/170 (39%)<br>Sex M/F: 35/31<br><br>**Non-Finnish patients:**<br>N=104/170 (61%)<br>Sex M/F: 44/104 (57.7%)<br><br><br>**Controls:**	**<u>Renal Transplantation (RTX):</u>**<br><br>N= /170 (88.8%)<br><br>Initial RRT:<br>Non-Finnish: N= 10/104 (9.9%)<br>CAKUT: N=66/312 (21.2%)<br><br><u>Median age at RTx:</u><br>Finnish NPHS1: 1.6 yrs (IQR 1.2-2.1)<br>Non-Finnish NPHS1: 3.0 yrs(IQR:1.7-4.4)<br>**P<0.01**<br><br><br>**<u>Dialysis / initial RRT:</u>**<br><br>Finnish: N=66/66 (100%) PD<br>Non-Finnish: N=69/104 (68.3%) PD<br>N=22/104 (21.8%) HD<br>CAKUT: N=188/312 PD<br>N=38/312 HD	**<u>Median age at start of RRT:</u>**<br><br>0.7 (ICR: 0.6-0.8) yrs (Finnish) vs 1.7 yrs (IQR: 1.0-2.9) other countries, **p<0.01**<br><br><br>**<u>5-year patient survival on RRT:</u>**<br><br><u>unadjusted</u><br>Finnish *NPHS1*: 91%<br>Non-Finnish *NPHS1*: 91%<br>CAKUT: 90%<br>P=0.83<br><br><u>Adjusted for age at the start of RRT and sex:</u><br><u>Risk of death (adjusted hazard ratio)</u><br>Finnish: 0.53 (95%CI: 0.21-1.33)<br>Non-Finnish: 0.84 (95%CI: 0.40-1.80)<br>CAKUT similar (no numbers)<br><br><br>**<u>5-year patient survival after first RTx:</u>**<br>Finnish: 98.4%

					Group of CAKUT patients age matched to non-finish patients<br>N=312<br>Sex M/F:251/61 (19.6%)	<u>Median Dialysis Time before RTx:</u> 0.9 (0.6-.7) years, no difference between two groups<br><br><u>Median age at start of RRT:</u><br>Finnish *NPHS1*: 0.7 (QR: 0.6–0.8) years<br>Non-Finnish *NPHS1*:1.7 (IQR: 1.0–2.9)<br>**P<0.01**	Non-Finnish: 97.8%<br>CAKUT control: 96.6%<br>P=0.42<br><br><u>Risk of death (adjusted hazard ratio) post RTx</u><br>Finnish: 0.32, 95 % CI: 0.04–2.86<br>Non-Finnish: 0.45, 95 % CI: 0.09–2.35<br>CAKUT control: similar (no numbers)<br><br>**<u>Renal Transplantation and Graft Survival</u>**<br><br><u>After 5 year follow-up:</u><br>graft survival did not differ between the groups: 89%, also comparable with 10 pre-emptive transplants (80%)<br><br>eGFR (ml/min/1.73m2):<br>Finnish: 54 (47-67)<br>Non-Finnish: 69 (59-83)<br>**P<0.01**<br>**→note that only 64% of Finnish and 24% of Non-Finnish patient were available**<br><br><u>After 8.5 year follow-up:</u><br>Grafts lost total N=26<br>Finnish: N=11 (18%)<br>Non-Finnish: N=15 (17%)
5	<u>Büscher A.</u>[5]<br><br><u>CJASN</u><br><br><u>(2016)</u><br><br><u>Germany</u><br><br>PMID:266680 27<br>PMCID: <u>PMC4741047</u><br>DOI: <u>10.2215/CJN. 07370715</u>	**Rapid Response to Cyclosporin A and Favorable Renal Outcome in Nongenetic Versus Genetic Steroid–Resistant Nephrotic Syndrome**	Retrospective Multicenter Study (Essen, Münster, Hannover, Köln, Innsbruck, Hamburg, Heidelberg, München))		Total N=231 (219 families)<br><br>Sex M/W: 106/215<br><br>N=62/231 (27%) CNS patients<br>*N=60/62 genetic*<br>*N=2/62 no variant detected*<br><br>N=169/231 (73%) primary SRNS<br>*N=71/169 genetic*<br>*N=98/169 non-genetic*<br><br>Median observation time:	**<u>CsA</u>**<br>Titrated to achieve blood level between 80 and 150 ng/ml<br><br><u>CNS patients:</u><br>N=0/2 (0%) non-genetic<br>N=9/56 (16%) genetic<br><br><u>SRNS patients:</u><br>N=82/96 (85%) non-genetic<br>N=32/68 (47%) genetic<br><br><u>Complete remission definition:</u><br>proteinuria ,4 mg/m2/h, urinary protein to creatinine ratio ,30 mg/mmol, or trace of protein on dipstick analysis and normalization of serum albumin (3.5 g/dl).	**<u>Genetic/Variant Analysis:</u>**<br>Overall detection rate: N=131/231 (57%)<br>*CNS: 97% N=60/62*<br>*SRNS: 42% N=71/169*<br><br>*NPHS1* (N=35), *NPHS2* (N=43), *WT1* (N=33) , *LAMB2* (N=3), *PLCE1* (N=2), TRPC6 (N=5), *INF2* (N=3), *ARHGDIA* (N=1), *LMX1B* (N=2)<br>Novel podocyte genes (N=2)<br><br>**<u>Response to CsA</u>**<br><u>CNS patients (genetic):</u><br>N=1/9 (11%) compound heterozygous NPHS1 variant) complete remission within 2 month and preserved normal renal function<br>N=8/9 (89%) no response<br><br><u>SRNS (genetic)</u>

					112 month (50-178 IQR)	Partial remission: definition proteinuria between 4 and 40 mg/m2/h and normalization of serum albumin (.3.5 g/dl).<br><br>**ACEi/ARB;**<br><br>CNS patients:<br>N=40/52 (77%) genetic (N=32 of these without concomitant CsA Rx)<br><br>SRNS patients:<br>N=80/94 (85%) non-genetic (N=10 of these without CsA)<br>N=51/64 (80%) genetic (N=23 of these without CsA)	N=1/32 (3%) complete remission within 2.5 month (ACTN4 variant)<br>N=5/32 (16%) partial remission after 1.5, 6 and 8 month<br>N=26/32 (81%)<br><br>SRNS (non-genetic)<br>N=49/81 (60%) complete remission (Median 2.5 month (ICR 1-5)<br>N=15/81 (19%) partial remission (Median 10.5 month (ICR:4-25)<br><br>**Renal Function:**<br>CNS patients (genetic):<br>N=50/60 (83%) median time to ESKD 24 month (7-40)<br>N=4/60 (7%) CKD<br>N=6/60 (10%) preservation of renal function<br><br>SRNS (genetic)<br>N=64/70 (66%) median time to ESKD 44 month (5-76)<br>N=13/23 (54%) ACEi/ARB Rx: ESKD<br>N=20/28 (71%) ACEi/ARB + CsA Rx : ESKD<br><br>N=6/70 (9%) CKD<br>N=18/70 (26%) preservation on renal function after 115 month follow-up (ICR 77-165)<br><br>SRNS (non-genetic)<br>N=69/96 (72%) normal renal function after media follow-up of 94 mo (41-159)<br>N=3/96 (3%) CKD<br>N=24/96 (25%) median time to ESKD 36 month (16-101)<br><br>**Transplantation**<br><br>CNS patients (genetic):<br>N=41/60 (68%) RTx without recurrence within observation time<br><br>SRNS (genetic)<br>N=41/71 RTx (58%)

							N=2/29 (7%) recurrence of the disease (no data N=12)<br><br>SRNS (non-genetic)<br>N=21/96 (22%) RTx<br>N=8/16 (50%) recurrence of disease (no date N=5)<br><br>**Renal Histology**<br><br>Non-genetic: FSGS 69% > MCD 23%.<br>Genetic: FSGS 21% (CNS) and 66% (SRNS), DMS 40% (CNS) and 14% (SRNS).
6	Büscher A.[6]<br><br>Clin J Am Soc Nephrol<br><br>(2010)<br><br>Germany<br><br>PMID:20798252<br>PMCID: PMC3001773<br>DOI: 10.2215/CJN.01190210	**Immunosuppression and Renal Outcome in Congenital and Pediatric Steroid-Resistant Nephrotic Syndrome**	Retrospective Study of all CNS and SRNS patients treated in Münster and Essen between 1999-2009		N=91 from 82 families<br><br>N=26/91 (29%) CNS<br><br>N=65/91 (71%) primary SRNS<br>*N=41 Non-genetic.*<br>*N=24 genetic*<br><br>Sex (M/F): 44/47<br><br>Mean Observation Time 103.0 ± 68.2 months	**CsA**<br>N=34 subsequent to CNS/SRNS diagnosis<br>*N=5/26 (19%) CNS patients*<br>*N=7/24 (29%) genetic SRNS*<br>*N=31/41 (76%) non-genetic SRNS*<br><br>mean dose at 6 months of therapy: 6.5±2.9 mg/kg/d<br><br>levels to achieve: 80 and 120 ng/ml<br><br>**ACEi/ARB:**<br><br>N=21/26 (81%) CNS patients<br>N=17/24 (71%) genetic SRNS<br>N=34/41 (83%) non-genetic SRNS<br><br>**Definitions**<br><br>Complete Remission:<br>Proteinuria <4 mg/m2/h or trace of protein on dipstick analysis and normalization of serum albumin (>3.5 g/dl)<br><br>Partial Remission:<br>Proteinuria between 4 and 40mg/m2/h and normalization of serum albumin.	**Genetic / Variant Analysis**<br>Variant detection rate 52%<br>(100% in CNS, 38% SRNS)<br>11 *NPHS1* (7 novel)<br>17 *NPHS2*<br>11 *WT1*<br>1 *LAMB2*<br>3 *TRPC6*<br><br>**Response to CsA :**<br>CNS: N=0/5 (0%), p<0.001 vs non-genetic form<br><br>SRNS (genetic): N=2/7 (29%) partial response (*WT1* variant), none showed complete response, p<0.09 vs non-genetic form<br><br>SRNS (non-genetic):<br>*N=17/31 (55%) complete remission*<br>*N=4/31 (13%) partial response*<br><br>**Development of ESRD:**<br>CNS: 84% p<0.0001 vs non-genetic<br><br>SRNS (genetic): : 58%, p<0.04 vs non-genetic<br><br>SRNS (non-genetic): 29%<br><br>**Mean Time to ESRD:**<br>CNS patients: 37.4 ± 27.6 months (1 died at age of 1 year to impaired renal function).<br>Genetic SRNS: 45.9 ± 45.2 month<br>Non-genetic SRNS: 50.1 47.0 months

7	Kovacevic L.[7]<br><br>Pediatr Nephrol<br><br>(2003)<br><br>USA<br><br>PMID:12687455<br>https://DOI: 10.1007/s00467-003-1131-3	**Management of Congenital Nephrotic Syndrome**	Retrospective study from 1990<br><br>Single-center (Guy´s Hospital London)	CNS<br><br>Captopril<br><br>Indomethacin<br><br>Unilateral nephrectomy<br><br>Management	N=7	**Unilateral Nephrectomy:**<br>N=7 (100%)<br>Median age: 2.6 month (2.1-31)<br><br>**Bilateral Nephrectomy+PD:**<br>N=4/7 (57%)<br>Median age: 36.5 month (31-40)<br>+Tx: N=4<br>Median age 54 month /42-72)<br><br>**Captopril + Indomethacin**<br>Commenced at median of 2.5 month of age (0.2-5.2 month)<br>Prior to UNx in 6 children<br>2 weeks after UNx in 1 child	**Median follow up: 54 month (36-88):**<br><br>Survival:<br>N=5/7 (71%) alive; median age 74 (43-88) month<br>N=2/7 (29%) died;<br>*N=1 age 48 mo; after 2nd nephrectomy, dialysis, transplantation*<br>*N=1 age 42 mo; E.coli peritonitis, septic shock, during nephrotic stage*<br><br>Plasma Albumin:<br>11 (6-17) g/l at start<br>18 (15-22) g/l after 6 months<br>21 (18-25) g/l after 12 months<br>P<0.001<br><br>Albumin infusion/patient/month:<br>7 (0-18) at start<br>0 (0-30) 6 months post treatment<br>P=0.017
8	Holmberg C.[8]<br><br>Pediatr Nephrol (1995)<br><br>Finland<br><br>PMID: 7742232 Feb;9(1):87-93	**Management of congenital nephrotic syndrome of the Finnish type.**	Review and single center study<br>Finnish Center 1985-1994	Nephrotic Syndrome Congenital Finnish Type Therapy	N=43 CNF		

Case Reports

9	Eichinger A.[9]<br>.<br>Pediatric Nephrology<br><br>(2018)<br><br>Germany	**Cyclosporine A responsive congenital nephrotic syndrome with single heterozygous variants in *NPHS1, NPHS2*, and *PLCE1***	Case Report	CNS<br><br>CsA<br><br>NPHS1<br><br>NPHS2<br><br>PLCE1	N=1 (F)<br><br>4-month old<br><br>Eritrean Origin<br><br>Symptoms at presentation:	Daily albumin infusion + furosemide, oral protein replacement vitamin and thyroid supplementation<br><br>**ACEi** (Captopril, max. 5mg/kg/d) and **indomethacin** (max. 5 mg/kg/d)<br><br>After genetic testing:<br>4-week **prednisolone** (60mg/m2/d)	**Genetics:**<br>WES: heterozygous variant in<br>NPHS1 (NM_004646.3: c.1219C > T, p.Arg407Trp)<br>NPHS2 (NM_014625.3: c.284C > T, p.Ser95Phe)<br>PLCE1(NM_016341.3: c.3133G > T, p.Ala1045Ser)<br><br>**Resistant to standard corticosteroid therapy**<br><br>**Cyclosporin A Rx:**

	https://doi.org/10.1007/s00467-018-3961-z				life-threatening anasarca, hypoalbuminemia, proteinuria, and impaired growth.<br><br>Kidney Biopsy:<br>Primary podocytopathy with minimal changes mesangial hypercellularity foot process effacement	**Daily cyclosporin A** (4mg/kg/d), oral magnesium substitution, ACEi 17 mg/d/d)	Within 3 month complete remission<br>i.v. albumin infusion discontinued<br>adverse effects:<br>mild CsA induced gingival enlargement and hypomagnesemia
10	Kim J. [10]<br><br>Pediatr Nephrol<br><br>(2011)<br><br>UK<br><br>https://doi10.1007/s00467-011-1911-0	**Nephrotic syndrome in infancy can spontaneously resolve**	Case Report	NS<br><br>Congenital<br><br>DMS<br><br>Pertussis<br><br>ACEi	Total N=4<br><br>Sex M/F: 2/2<br>Caucasians<br>No consanguinity<br>No dysmorphia<br><br>CNS patients:N=2 (M)<br><br>15 days – 7 months<br><br>Infectious and genetic tests negative	Albumin infusion + furosemide + ACE/ARBs + Penicillin V<br>1 received steroids	Duration of albumin therapy: 2 days – 4 months<br>Age at resolution of NS: 3.5 – 13 months<br>Age at stopping all medications: 5 – 30 months<br><br>Normal function and urinary sediment at last follow-up : 3.5 – 13 years
11	Slaughenhoupt BL.<br><br>Urology<br><br>(1998)<br><br>USA	**Urologic Management of Congenital Nephrotic Syndrome of the Finnish Type**	Case Report		N=1 (F)<br><br>7 mo of age		Successful bilateral nephrectomy and transplantation in an infant with refractory CNS.

Genetics

No	1st author, year, country of origin [Ref.]	Title of Publication	Study design	Key-words	Patients	Intervention and comparator	Outcomes
12	Braun D.[11]<br><br>American Journal of Medical Genetics<br><br>(2018)<br><br>USA<br>https://DOI:10.1002/ajmg.a.40489	**Mutations in *WDR4* as a new cause of Galloway–Mowat syndrome**	Clinical Report	Galloway-Mowat-Syndrome<br><br>Mendelian Diseases<br><br>Rare syndromic diseases<br><br>Variable phenotypic expressivity<br><br>WES	N=4 children from Indian family with GAMOS	**Clinical characteristic**<br>N=4/4 (100%) : Microcephaly, global development delay, variable degrees of intellectual disability, growth retardation<br><br>N=2/4 (50%) Clinodactyly<br><br>N=3/4 (75%) renal involvement<br>Nephrotic range proteinuria<br>Variation of severity<br>Only one child showed complete symptoms of NS	**Whole Exome Sequencing:**<br>N=3 children with renal involvement<br>Homozygous variant in WDR4 gene→ cause recessive GAMOS
13	Braun D.[12]<br><br>Nat Genet<br><br>(2017)<br><br>USA<br><br>https://doi:10.1038/ng.3933	**Mutations in the evolutionarily highly conserved KEOPS complex genes cause nephrotic syndrome with microcephaly**	Clinical Report	Galloway-Mowat-Syndrome<br><br>KEOPS	N=91 patients with GAMOS	**Clinical characteristic**<br>Microcephaly, developmental delay, propensity for seizures, early onset NS<br><br>Renal biopsy:<br>FSGS, DMS;<br>Partial foot process effacement<br><br>No response to steroids or CsA (only in N=3 therapeutic attempt)	**Whole Exome Sequencing:**<br>N=33/91 (34%) recessive mutations in KEOPS genes<br>30 different families:<br>*N=22/30: OSGEP (15 alleles)*<br>*N=3/30: TP53RK (4 alleles)*<br>*N=2/30 :TPRKB (2 alleles)*<br>*N=3/30: LAGE3 (3 alleles)*
14	Cil O.[13]<br><br>Pediatr Nephrol<br><br>(2015)<br><br>Turkey	**Genetic abnormalities and prognosis in patients with congenital and infantile nephrotic syndrome**	Phenotype-Genotype Correlation	CNS<br><br>Infantile nephrotic Syndrome<br><br>NPHS1<br>NPHS2	N=80 CNS patients<br><br>N=22 INS	**Clinical characteristic**<br>Edema,<br>Proteinuria<br>Microscopic hematuria (60% of patients with *NPHS1* or *NPHS2* variants)	**Sanger Sequencing**

	https://DOI10.1007/s00467-015-3058-x			WT1<br>LAMB2			
15	Binczak-Kuleta A.<br><br>Bosn J Basic Med Sci<br><br>(2014)<br><br>Poland<br><br>PMID: 24856380<br><br>PMCID: PMC4333957<br>DOI: 10.17305/bjbms.2014.2270	**Retrospective mutations analysis of *NPHS1, NPHS2, WT1,* and *LAMB2* in children with steroid-resistant focal segmental glomerulosclerosis- a single center experience**	Retrospective study in polish patient cohort	Gene variant<br><br>NPHS2<br><br>WT1<br><br>Children<br><br>SRNS<br><br>FSGS	N=33 NS patients<br>*N=7/33 (21%) onset before 1st year*<br>*N=15/33(45%) early childhood onset (13-60 month)*<br>*N=11/33 (33%) late childhood-onset (61-132 month)*<br><br>Sex M/F:15/18	**Clinical Characteristics**<br>Biopsy proven FSGS<br><br>Primary FSGS (8 weeks of daily prednisone)<br><br>Persistence of heavy proteinuria (>50mg/kg/d)	No pathogenic mutation found in *NPHS1* or *LAMB2*<br><br>***NPHS2* gene variants**<br>Prevalence<br>Overall: N=8/33 (24%)<br><br>Early onset (>12 month) N=5/8 (62%%)<br><br><br>***WT1* gene variant**<br>Prevalence<br>Overall N=2/33 (6%)<br><br>Early onset: N=1/2 (50%)
16	Abib A.[14]<br><br>Gene<br><br>(2012)<br><br>Pakistan<br><br>https://doi:10.1016/j.gene.2012.04.063	**A spectrum of novel NPHS1 and NPHS2 gene mutations in pediatric nephrotic syndrome patients from Pakistan**<br>Supplementary data to this article can be found online at https://doi:10.1016/j.gene.2012.04.063<br><br>.	Comprehensive Screening of *NPHS1* and *NPHS2* gene mutations in pediatric NS case in South Asia (Pakistan)	CNS<br><br>FSGS<br><br>NPHS1<br><br>NPHS2<br><br>Podocyte<br><br>SRNS	N=145 NS patients<br><br>Sex M/F: 88/57<br>*N=36/145 (25%) early-onset (congenital and infantile)*<br><br>*N=109/145 (75%) childhood-onset*<br><br>N=39/145 (27%) familial cases (30 families)	**Clinical Characteristics**<br><br>FSGS 8%, MCD 27%, IgMN 9%, MesPGN 9%, MN 3%, others 3%<br><br>ESRD 9.6%	***NPHS1***<br><br>prevalence<br>overall N=8/145 (5.5%)<br>7 homozygous variants found (6 novel ones)<br>Early-onset cases N=8/36 (22%):<br>Homozygous or compound heterozygous variants:<br>No remission: p.R1160X, p.T1182A, p.L237P, p.A912T, p.G867P.<br>Partial remission to CsA : p.G1020V.<br><br>Heterozygous variants: 2 no remission, 6 partial remission, 1 complete remission, 3 maintained on ACEi+ARB<br><br>***NPHS2***<br>prevalence<br>overall N=5/145 (3.4%)<br>4 homozygous variants found: p.R229Q, p.P341S, p.K126N, p.V260E<br>Early onset cases N=2/36 (5.5%)<br>1 no remission, 2 partial remission, 2 maintained on ACEi+ARB

Post-Tx

No	1st author, year, country of origin [Ref.]	Title of Publication	Study design	Key-words	Patients	Intervention and comparator	Outcomes
17	Guler S.[15]<br><br>Pediatr Transplant<br><br>(2017)<br><br>Canada<br><br>PMID: 29094445<br>https://doi:10.1111/petr.13076	**Kidney transplantation in a child with Pierson syndrome**	Case report (1 patient) with Pierson syndrome *(LAMB2* heterozygous)<br><br>Department of Pediatrics, University, Halifax, Canada	congenital nephrotic syndrome<br><br>kidney transplantation,<br>microcoria<br><br>Pierson syndrome	N=1	RTx	→A case of Pierson syndrome successfully treated with deceased donor kidney transplantation<br><br>→Pierson syndrome should be considered in children with renal manifestations and ocular abnormalities.<br><br>→ Children with Pierson syndrome must be evaluated in terms of kidney transplantation as soon as they are diagnosed
18	Hölttä T.[4]<br><br>Pediatr Nephrol<br><br>(2016)<br><br>Finland<br><br>PMID: 27761660<br>https://DOI:10.1007/s00467-016-3517-z	**Timing of renal replacement therapy does not influence survival and growth in children with congenital nephrotic syndrome caused by mutations in *NPHS1*: data from the ESPN/ERA-EDTA Registry**	Retrospective study<br><br>Data from Jan 1991 – 31 Dec 2012 collected<br><br>Within ERA-EDTA/ESPN registry	Congenital nephrotic syndrome<br>Dialysis<br>Graft survival;<br>Kidney transplantation; NPHS1<br>Pediatrics	Total N=170 CNS patients<br><br>100% NPHS1 mutations<br><br>initiated RRT<br><br>Sex M/F: 79/91<br><br>**Finnish patients:**<br>N=66/170 (39%)<br>Sex M/F: 35/31<br><br>**Non-Finnish patients:**<br>N=104/170 (61%)<br>Sex M/F: 44/104 (57.7%)	<u>**Renal Transplantation:**</u><br><br>N=150/170 (88.8%)<br><br>Initial RTx:<br>Non-Finnish: N= 10/104 (9.9%)<br>CAKUT: N=66/312 (21.2%)<br><br><u>Median age at RTx:</u><br>Finnish *NPHS1*: 1.6 yrs (IQR 1.2-2.1)<br>Non-Finnish *NPHS1*: 3.0 yrs (IQR:1.7-4.4)<br>**P<0.01**<br><br><u>**Dialysis / initial RRT modality:**</u><br><br>Finnish: N=66/66 (100%) PD<br>Non-Finnish: N=69/104 (68.3%) PD<br>N=22/104 (21.8%) HD	<u>**Median age at start of RRT:**</u><br>Finnish *NPHS1*: 0.7 (QR: 0.6–0.8) years<br>Non-Finnish *NPHS1*:1.7 (IQR: 1.0–2.9)<br>**P<0.01**<br><br><u>**Median Dialysis Time before RTx:**</u> 0.9 (0.6-.7) years, no difference between two groups<br><br><u>**5-year patient survival on RRT:**</u><br><br><u>unadjusted</u><br>Finnish NPHS1: 91%<br>Non-Finnish NPHS1: 91%<br>CAKUT: 90%<br>P=0.83<br><br><u>Adjusted for age at the start of RRT and sex:</u>

					 <br>**Controls:**<br>Group of CAKUT patients age matched to non-finish patients<br>N=312<br>Sex M/F:251/61 (19.6%)	CAKUT: N=188/312 PD<br>N=38/312 HD	<u>Risk of death (adjusted hazard ratio)</u><br>Finnish: 0.53 (95%CI: 0.21-1.33)<br>Non-Finnish: 0.84 (95%CI: 0.40-1.80)<br>CAKUT similar (no numbers)<br><br>**<u>5-year patient survival after first RTx:</u>**<br>Finnish: 98.4%<br>Non-Finnish: 97.8%<br>CAKUT control: 96.6%<br>P=0.42<br><br><u>Risk of death (adjusted hazard ratio) post RTx</u><br>Finnish: 0.32, 95 % CI: 0.04–2.86<br>Non-Finnish: 0.45, 95 % CI: 0.09–2.35<br>CAKUT control: similar (no numbers)<br><br>**<u>Renal transplantation and graft survival</u>**<br><br><u>After 5 year follow-up:</u><br>graft survival did not differ between the groups: 89%, also comparable with 10 pre-emptive transplants (80%)<br><br>eGFR (ml/min/1.73m2):<br>Finnish: 54 (47-67)<br>Non-Finnish: 69 (59-83)<br>**P<0.01**<br>**→note that only 64% of Finnish and 24% of Non-Finnish patient were available**<br><br><u>After 8.5 year follow-up:</u><br>Grafts lost total N=26<br>Finnish: N=11 (18%)<br>Non-Finnish: N=15 (17%)
19	Martínez Mejía S.[16]<br><br>Transplant Proc.<br><br>2015<br><br>Nefrología	**Renal Transplantation in Children With Nephrotic Syndrome in the First Year of Life**	Retrospectiv study<br>With NSFL receiving transplant<br>1989-2303	NS in the 1st year of life (NSFL)underwent RTx	N=15<br><br>Sex M/W: 8/7<br><br>N=9/15 (60%) finnish type<br><br>N=4/15 (27%) DMS	**mean age at diagnosis**:<br>2.21 month (0-8.2 months);<br><br>**mean age at onset of RRT**:<br>22.9 month (3.8-55.4 month)<br><br><br>**mean age on dialysis:**<br>**14.9 month(2-44 month)**	-After a **<u>mean follow-up time of 72.8 month</u>** <u>(</u>range, 1 month to 16.9 years) from first transplantation, we found an actuarial survival at both 1 and 7 years of 92.9%, which is similar to that observed in others series<br><br>-1case of acute rejection 1 month after transplantation, and 2 for chronic rejection >9 years after transplantation

	Infantil La Paz,<br>Castellana<br>261, 28046,<br>Madrid,<br>Spain<br>PMID:<br>25645765<br>https://DOI10.1016/j.transproceed.2014.11.022				N=1/15 (7%) MCGN<br><br>N=1/15 (7%)<br>Collapsing<br>Glomerulopathy	**Age at transplantation:**<br>**3.1 yrr (1.8-7.7 yrs)**<br><br>N=6 LDTs<br>N=9 CDTs<br><br>N=10 patients required<br>pre-transplantation nephrectomy (9<br>bilateral) to control<br>proteinuria after a mean time on<br>dialysis of 3.1 _ 3.8<br>months;<br><br>There were 3 graft losses, all<br>from CD: 1 acute rejection 1 month<br>after transplantation,<br>and 2 chronic rejections >9 years after<br>transplantation.<br><br>Neither case had recurrence of the<br>underlying disease.	-short-term complications did not differ<br>from the rest of the population of transplant<br>children if surgery is performed with normal<br>albumin levels
20	Holmberg<br>C.[17]<br><br>Pediatr<br>Nephrol<br><br>(2014)<br><br>Finnland<br><br><br>PMID:<br>24682440<br>DOI:10.1007/s00467-014-2781-z	Congenital nephrotic<br>syndrome and recurrence<br>of proteinuria<br>after renal<br>transplantation	Review focused<br>on the<br>rare cases of<br>proteinuria<br>recurrence in<br>patients with<br>genetic causes<br>of CNS after<br>renal RTx.<br><br>Children's<br>Hospital,<br>University of<br>Helsinky	Congenital<br>nephrotic<br>syndrome .<br><br>Renephrosis .<br><br>-Anti-<br>nephrin<br>antibodies<br><br>-Rituximab		**<u>1)Recurrent proteinuria in NPHS1 patients</u>**<br><br>→**<u>Laine et al</u>** (1992)<br>28 CNF patients<br>6 (24 %) developed severe<br>proteinuria and NS 1-33 months<br>After RTx<br>-treated with methylprednisolone<br>(MP) and 5 with additional<br>cyclophosphamide (CP).<br>- 2 patients remission<br>4 grafts were lost.<br>1 showed proteinuria again in<br>the second graft 14 months after RTx<br><br>→**<u>Patrakka</u>** (2000)<br>45 CNF (NPHS1) patients<br>receiving 51 kidneys<br>15recurrence proteinuria in 13 grafts<br>(25 %).<br>-treated with CP was successful in 7 p,<br>but 6 kidney were lost.<br>-Antibodies reacting against the<br>glomerulus in 8 out of 9 patients<br>-Serum antinephrin antibody in 4p	 <br><br><br>→→**<u>Laine et al</u>** (1992) : a risk for proteinuria in<br>CNF seems to exist after early RTx,<br>with some patients responding to therapy.<br><br><br><br><br><br><br><br><br><br><br><br>→→ **<u>Patrakka</u>** (2000) : circulating anti-nephrin<br>antibodies play a pathogenic role in NS<br>recurrence. This resembles the<br>situation in Alport's syndrome, in which an<br>immune response against the previously unseen<br>collagen epitopes in kidney<br>grafts is responsible for the de-novo anti-<br>glomerular basement membrane disease

						→ **Kuusniemi et al. (2007)**<br>65 Finnish CNF patients<br>who had received 77 kidneys<br>-recurrence rate in Finmajor/ Fin-major patients was then 34 %, with anti-nephrin antibodies found in 8/11 patients (73 %)<br>- In patients with a re-transplantation, recurrence occurredin 1–22 days<br>- Plasma exchange (PE) in<br>MP and CP in 7 patients NS after transplantation. Only one graft was lost (11 %) in contrast to five (45 %) in the 11 patients treated only with steroids and CP.<br>PE for 5 days and then 3 times a week depending on response a.<br>-CP was given for 12 weeks and then switched to AZA or MMF as part of triple immunosuppression.<br>- 1 patient failing to react to PE also received Rituximab and high-dose immunoglobulin<br>without any response.<br><br>→**Srivastava (2006)**<br>-1 patient with NS after 7 days RTx<br>- No anti-nephrin antibodies were present, but as serum permeability activity was high<br><br>→ **Stanford (2012)**<br>-1 patient RTx who developed NS with anti-nephrin antibodies 6 months after transplantation biopsy: slight rejection and CD20-staining was positive.<br>-CP, steroids + Rituximab (remission)<br><br>→**Holmberg**<br>-6 patient reccurent NS<br>-high dose MP,CS,PE<br>-5 patients Rituximab<br>-2 patinents Rit + Bortezomib<br>6 out of 7 remssion	→→ **Kuusniemi et al. (2007)** the recurrence rate is about 30 % in Fin-major/Fin-major patients, that 70 % have measurable anti-nephrin antibodies, and that most<br>patients react to MP and CP combined with PE, and their GFR remains good.<br><br>→→ **Srivastava (2006)** it suggested a circulating factor might play a role in re-nephrosis<br><br>→→ **Stanford (2012)** Rituximab might<br>have contributed to that favorable result.

						**<u>2)Recurrent proteinuria in NPHS2 patients</u>**<br><br>→**<u>Bertelli et al.</u>**<br>-2 patient with recurrence of proteinuria after RTx<br>-treated PE + CP<br><br>**<u>→ Billing et al.</u>**<br>-1 patient with recurrence of proteinuria after RTx<br>-treated PE + CsA<br><br>**<u>→Becker-C ohen</u>**<br>-1 patient with recurrence of proteinuria after RTx<br>-treated PE<br><br>**<u>→Weber et al.</u>**<br>-1 ou t of 32 patients with FGSG 2 yrs after RTx<br><br>**<u>→Höcker et al</u>**.<br>-1 patient with FSGS 10 years post-RTx in association with conversion from CsA-based to sirolimus-based immunosuppression.	→→ **<u>Bertelli et al.</u>** Good outcome<br><br><br><br><br>→**<u>→ Billing et al.</u>** maintained stable graft function and no recurrence of proteinuria has been observed.<br><br><br><br><br><br><br><br><br>**<u>→→Weber et al.</u>**No anti-podocin antibodies appeared in indirect immunofluorescence.<br><br><br>**<u>→→Höcker et al</u>**. A re-switch of the immunosuppressive regimen back to CsA led to a noticeable decrease in proteinuria and tostabilization of graft function<br><br><br>**<u>→Review Key summary points</u>**<br>- Severe proteinuria and NS are very rare in primary CNS patients after RTx<br>- The most probable etiology is NPHS1 with a gene variant leading to absence of nephrin, as occurs in Fin-major homozygotes<br>- In the majority of these patients, anti-nephrin antibodies are detectable after RTx<br>- Sometimes NS can develop after RTx in patients with NPHS2 variants, but the mechanisms are still elusive<br>- The best treatment options known today are MP, cyclophosphamide, and plasma exchange alone or combined with<br>Rituximab

21	Chaudhuri A[18]<br><br>Pediatr Transplant<br><br>(2012)<br><br>Departments of Pediatrics and Surgery, Stanford University, Stanford, CA, USA<br><br>PMID: 21672106<br>https://DOI:10.1111/j.13993046.2011.01519.x	**Rituximab treatment for recurrence of nephrotic syndrome in a pediatric patient after renal transplantation for congenital nephrotic syndrome of Finnish type**	Case report (1 patient)<br><br>→Genetic testing for nephrin revealed a homozygous (NPHS1)	rituximab<br><br>recurrence of nephrotic Syndrome<br><br>pediatric transplantation	N=1	**Rituximab:**<br>4 doses of 375 mg/m2/dose at weekly intervals<br><br>combination with<br>- cyclophosphamide,<br>- corticosteroids,<br>- tacrolimus,<br>- MMF	→A case of post-transplant recurrence of NS in a child with CNS owing to NPHS-1<br><br>→Precipitous fall in anti-nephrin antibodies following RTX therapy also coincided with clinical response.<br><br>→RTX, which may have played the pivotal role in achieving and sustaining remission, suggesting that RTX may be considered in cases like this who are resistant to standard therapy.<br><br>→The child was treated with a variety of immunosuppressive agents including RTX, which may have played the pivotal role in achieving and sustaining remission, suggesting that RTX may be considered in cases like this who are resistant to standard therapy.

ACEi : angiotensin-converting enzyme inhibitors, ARBs : angiotensin type I receptor blockers, CAKUT: congenital anomalies of the kidney and urinary tract, CCPD: continuous cycling peritoneal **dialysis**, CKD : chronic kidney disease, CNF: congenital nephrotic syndrome of the Finnish type, CNS : congenital nephrotic syndrome, CsA : cyclosporin A, CVVH : continuous veno-venous hemofiltration, DMS : diffuse mesangial sclerosis, eGFR: estimated glomerular filtration rate, ERA-EDTA : European Renal Association – European Dialysis and Transplantation Association, ESKD : end-stage kidney disease, ESPN : European Society of Paediatric Nephrology, FSGS : focal segmental glomerulosclerosis, GAMOS: Galloway-Mowatt syndrome, HD: hemodialysis, IgMN: IgM Nephropathy, IV: intra-venous, IVIG : intra-venous immunoglobulins, (M/F): Male/Female, MesPGN: Mesangial Proliferative Glomerulonephritis, MN: Membranous Nephropathy, mo: month, NX : nephrectomy, NS: nephrotic syndrome, n.s.: not significant, PD: peritoneal dialysis, S-Albumin: serum albumin level, RRT : renal replacement therapy, RTx : renal transplantation, Rx : Treatment, SRNS : steroid-resistant nephrotic syndrome, Tx : transplantation, UNx: unilateral nephrectomy, WES: whole exome sequencing, yr : year.

REFERENCES

1. Berody, S. *et al.* Treatment and outcome of congenital nephrotic syndrome. *Nephrol Dial Transplant* **34**, 458–467 (2019).
2. Dufek, S. *et al.* Management of children with congenital nephrotic syndrome: challenging treatment paradigms. *Nephrol Dial Transplant* (2019) doi:10.1093/ndt/gfy165.
3. Dufek, S. *et al.* Infants with congenital nephrotic syndrome have comparable outcomes to infants with other renal diseases. *Pediatr Nephrol* (2019) doi:10.1007/s00467-018-4122-0.
4. Holtta, T. *et al.* Timing of renal replacement therapy does not influence survival and growth in children with congenital nephrotic syndrome caused by mutations in NPHS1: data from the ESPN/ERA-EDTA Registry. *Pediatr Nephrol* **31**, 2317–2325 (2016).
5. Buscher, A. K. *et al.* Rapid Response to Cyclosporin A and Favorable Renal Outcome in Nongenetic Versus Genetic Steroid-Resistant Nephrotic Syndrome. *Clin J Am Soc Nephrol* **11**, 245–53 (2016).
6. Buscher, A. K. *et al.* Immunosuppression and renal outcome in congenital and pediatric steroid-resistant nephrotic syndrome. *Clin J Am Soc Nephrol* **5**, 2075–84 (2010).
7. Kovacevic, L., Reid, C. J. & Rigden, S. P. Management of congenital nephrotic syndrome. *Pediatr Nephrol* **18**, 426–30 (2003).
8. Holmberg, C., Antikainen, M., Ronnholm, K., Ala Houhala, M. & Jalanko, H. Management of congenital nephrotic syndrome of the Finnish type. *Pediatr Nephrol* **9**, 87–93 (1995).
9. Eichinger, A. *et al.* Cyclosporine A responsive congenital nephrotic syndrome with single heterozygous variants in NPHS1, NPHS2, and PLCE1. *Pediatr Nephrol* **33**, 1269–1272 (2018).
10. Kim, J. J. *et al.* Nephrotic syndrome in infancy can spontaneously resolve. *Pediatr Nephrol* **26**, 1897–901 (2011).
11. Braun, D. A. *et al.* Mutations in *WDR4* as a new cause of Galloway-Mowat syndrome: Mutations in *WDR4* as a new cause of Galloway-Mowat syndrome. *American Journal of Medical Genetics Part A* **176**, 2460–2465 (2018).
12. Braun, D. *et al.* Mutations in KEOPS-complex genes cause nephrotic syndrome with primary microcephaly. *Nat Genet* **49**, 1529–1538 (2017).
13. Cil, O. *et al.* Genetic abnormalities and prognosis in patients with congenital and infantile nephrotic syndrome. *Pediatr Nephrol* **30**, 1279–87 (2015).
14. Abid, A. *et al.* A spectrum of novel NPHS1 and NPHS2 gene mutations in pediatric nephrotic syndrome patients from Pakistan. *Gene* **502**, 133–137 (2012).
15. Guler, S., Cimen, S., Acott, P., Whelan, K. & Molinari, M. Kidney transplantation in a child with Pierson syndrome. *Pediatr Transplant* **21**, (2017).
16. Martínez Mejía, S. *et al.* Renal transplantation in children with nephrotic syndrome in the first year of life. *Transplant Proc* **47**, 38–41 (2015).
17. Holmberg, C. & Jalanko, H. Congenital nephrotic syndrome and recurrence of proteinuria after renal transplantation. *Pediatr Nephrol* **29**, 2309–17 (2014).
18. Chaudhuri, A. *et al.* Rituximab treatment for recurrence of nephrotic syndrome in a pediatric patient after renal transplantation for congenital nephrotic syndrome of Finnish type. *Pediatr Transplant* **16**, E183-7 (2012).
